# A Systematic Review of Genotype–Phenotype Correlation across Cohorts Having Causal Mutations of Different Genes in ALS

**DOI:** 10.3390/jpm10030058

**Published:** 2020-06-29

**Authors:** Owen Connolly, Laura Le Gall, Gavin McCluskey, Colette G Donaghy, William J Duddy, Stephanie Duguez

**Affiliations:** 1 Northern Ireland Center for Stratified/Personalised Medicine, Biomedical Sciences Research Institute, Ulster University, Londonderry BT47 6SB, Northern Ireland, UK; Connolly-O4@ulster.ac.uk (O.C.); Le_Gall-L@ulster.ac.uk (L.L.G.); gmccluskey05@qub.ac.uk (G.M.); w.duddy@ulster.ac.uk (W.J.D.); 2 Department of Neurology, Altnagelvin Hospital, WHSCT, Londonderry BT47 6SB, Northern Ireland, UK; ColetteG.Donaghy@westerntrust.hscni.net; 3 Motor Neurone Disease Care Centre, Royal Victoria Hospital, Belfast BT12 6BA, Northern Ireland, UK

**Keywords:** ALS, MND, ALS variants, genotype–phenotype, ALS genes

## Abstract

Amyotrophic lateral sclerosis is a rare and fatal neurodegenerative disease characterised by progressive deterioration of upper and lower motor neurons that eventually culminates in severe muscle atrophy, respiratory failure and death. There is a concerning lack of understanding regarding the mechanisms that lead to the onset of ALS and as a result there are no reliable biomarkers that aid in the early detection of the disease nor is there an effective treatment. This review first considers the clinical phenotypes associated with ALS, and discusses the broad categorisation of ALS and ALS-mimic diseases into upper and lower motor neuron diseases, before focusing on the genetic aetiology of ALS and considering the potential relationship of mutations of different genes to variations in phenotype. For this purpose, a systematic review is conducted collating data from 107 original published clinical studies on monogenic forms of the disease, surveying the age and site of onset, disease duration and motor neuron involvement. The collected data highlight the complexity of the disease’s genotype–phenotype relationship, and thus the need for a nuanced approach to the development of clinical assays and therapeutics.

## 1. Introduction

Amyotrophic lateral sclerosis, or ALS, is characterised by a progressive and fatal degeneration of upper and/or lower motor neurons (UMN and LMN, respectively) resulting in muscle weakness and wasting. Classical ALS is the most common form of motor neuron disease (MND) [1] and is defined by the selective deterioration of both UMN and LMN [2]. The global incidence of ALS varies between 1 and 2.6 cases per 100,000 people per year [3], with the average age of onset ranging from 54 to 67 years old [4]. The prevalence of ALS increases with age, reaching 1/5000 among people aged 70–79 years old [5]. Consequently, as the population ages, it is expected that the world’s total number of cases will reach more than 375,000 by 2040 [6]. Owing to the lack of a reliable diagnostic test, absence of validated biomarkers, and phenotypes that are easily confounded with other MNDs, including primary lateral sclerosis (PLS) and progressive muscular atrophy (PMA), there is a delay of approximately 11–12 months in reaching a definite diagnosis [7]. Currently, diagnosis is based on a set of clinical criteria (El Escorial [8] and revisions [9], and Awaji-Shima criteria [10]) that can be used to stratify patients according to the area of initial onset and the progression of symptoms.

**Figure 1:**
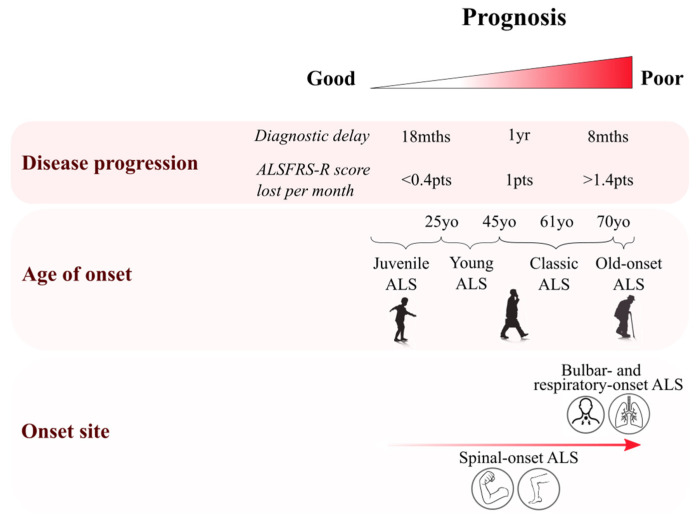
Clinical features of amyotrophic lateral sclerosis (ALS) and their role in prognosis. Diagram summarising the heterogeneity of clinical features in ALS. Multiple features have been associated with a poor prognosis, with an elderly onset being associated with a rapid progression of symptoms and a poor prognosis, especially among elderly females presenting with bulbar-onset phenotype [12]. Disease progression can be assessed either by diagnostic delay or by the ALS functional rating score (ALSFRS: amyotrophic lateral sclerosis functional rating scale). Poor prognosis is associated with patients whose ALS diagnosis has been given less than 8 months after symptom onset, or among those patients losing more than 1.4 points/month on the ALSFRS scale [13].

ALS phenotypes vary between patients who can present with different sites of onset and symptom severity (Figure 1). Concomitant impairments in cognitive ability are sometimes associated with the ALS phenotype. A recent finding from Chiò et al. suggested that 20.5% of ALS patients had frontotemporal dementia (FTD), and a further 31.3% had a behavioural, cognitive or non-executive impairment [11].

In the past 30 years, there have been a large number of studies investigating the genetic underpinnings of ALS. To date, over 30 genes have been related to the disease; yet it is important to note that mutations in these genes explain only ~20% of total ALS cases [14] whilst the majority of cases remain unexplained and present no family history. ALS is therefore considered to be a mainly sporadic disease (sALS), with ~80% of cases having no known genetic basis [3], although twin studies have estimated heritability at 40–45% [15] or 61% [16]. Known gene mutations explain some 70% of familial cases (fALS) [17,18], and they have also been identified in 10% of sporadic cases [18]. In European cohorts, the hexanucleotide repeat expansion in the *C9orf72* gene is the most common genetic cause of fALS (33.7%) and sALS (5.1%), followed by *SOD1* (14.8% in fALS and 1.2% in sALS cases), *TARDBP/TDP-43* (4.2% in fALS and 0.8% in sALS), and *FUS* (2.8% in fALS and 0.3% in sALS) [19].

To understand the molecular mechanisms underlying ALS, it is useful to study genotype–phenotype relationships, to determine whether certain gene mutations are associated with specific clinical features or outcomes. Genotype–phenotype relationships have previously been examined for certain gene mutations, and several informatics resources exist to collect genotype–phenotype data [20,21,22,23,24], but a systematic understanding across different gene mutations has not been established. As a step towards this, the present review gathers together the clinical summary statistics from previously studied cohorts across 22 of the more commonly associated genes. Each of the genes are considered in the order in which they were discovered and, where available, a summary of the reported phenotypes associated with each gene is later provided.

## 2. Pathological Definition of ALS: Clinical Features and Phenotype Variability

### 2.1. Age of Onset Variation

ALS occurs primarily in patients in their sixth decade, though peak onset is later in sporadic cases (58–63 years) than in familial cases (47–52 years) [25] (Table 1 and Figure 1). Four periods of onset can be defined: juvenile (<25 years old); young (25–45 years); mid–late adulthood (45–70 years); and elderly (>70 years). Juvenile ALS is extremely rare (<1/1,000,000 cases) [26], and is usually associated with slower symptom progression, hence a longer survival time and better prognosis [27]. Some mutations are now described to be associated with juvenile ALS, such as specific mutations of *FUS*, *ALS2* and *SETX* genes [26]. UMN rather than LMN dysfunction is predominant among juvenile ALS cases. Young-onset ALS also shows mainly UMN dysfunction, which is predominant in 60% of those patients [26]. Bulbar-onset ALS is rare in young patients and represents ~15% of cases [27]. In addition, young-onset ALS affects a relatively high proportion of males, with a male:female ratio of 3:1 [26]. These young-onset cases are also associated with a better prognosis than older ALS patients. Elderly-onset patients are more likely to present with bulbar symptoms and are represented by a greater proportion of female patients (M:F ratio 1–1.6) [12,26]. Symptom onset after 80 years is associated with a more aggressive phenotype and poor prognosis, with mean survival times of less than 20 months [12].

### 2.2. Site of Onset Variability

The majority of ALS cases (~70%) have spinal onset, usually presenting with focal limb weakness [30] such as foot drop or a weak hand [7]. The disease then tends to spread in a contiguous manner, initiating at distinct focal regions of the body and then propagating from the primarily affected area to adjuvant secondary sites of the body [31].

In 25% of ALS cases, symptoms develop initially in the bulbar-innervated muscles [30,32]. Bulbar-onset ALS is more common in women [7], especially after 70 years (M:F ratio 1:1.6 [12]). Dysarthria almost always predates dysphagia and cognitive impairment is often present [32].

Approximately 3% to 5% of patients [33] present with respiratory or cognitive onset [25]. Thoracic spinal-onset ALS can present as truncal weakness or respiratory impairment and is associated with poor prognosis, with a mean survival time of just 1.4 years [27,34].

Cognitive-onset ALS patients usually present symptoms characteristic of frontotemporal dementia (FTD), such as changes in behaviour, personality and cognition which are all suggestive of frontal impairments [35].

In summary, initial site of symptom onset varies among ALS patients from classic limb-onset to rare cognitive-onset phenotypes, and a poor prognosis is often associated with bulbar and respiratory onset [25].

### 2.3. Motor Neuron Involvement in ALS Variants

**Figure 2:**
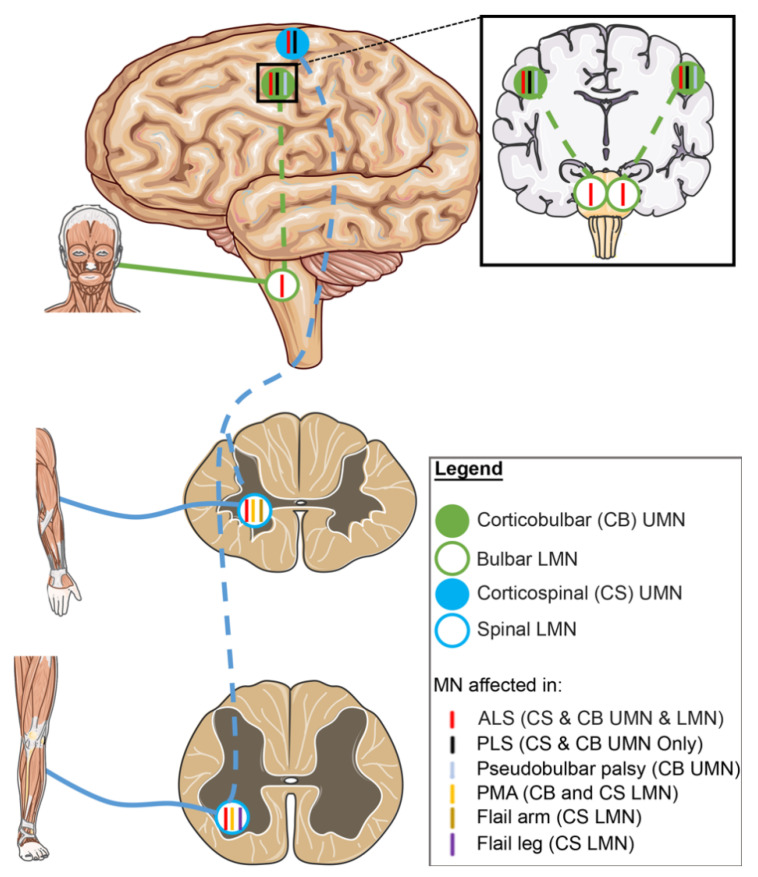
The role of upper and lower motor neurons in different ALS variants. ALS is a disease with high variability in clinical phenotype. “Classic ALS” patients will present with signs of both UMN and LMN degeneration. However, patients with progressive muscular atrophy (PMA) and primary lateral sclerosis (PLS) present with LMN-predominant or UMN-predominant signs, respectively. LMN-predominant patients also include flail-arm syndrome and flail-leg syndrome ALS variants where LMN signs are present in upper or lower limbs, respectively. ALS patients might present symptoms in bulbar-innervated muscles, if UMN signs are predominant, patients are diagnosed with pseudobulbar-palsy. Blue colour circles indicate motor neurons of the corticospinal tract. Green colour circles indicate motor neurons of the corticobulbar tract. Solid circles indicate UMNs and open circles indicate LMNs. Colour of ticks corresponds to colour of variant label and tick location indicates the motor neuron populations affected. ALS: amyotrophic lateral sclerosis. PLS: primary lateral sclerosis. PMA: progressive muscular atrophy. CS: corticospinal. CB: corticobulbar.

ALS patients can present with either a LMN or UMN predominant phenotype (Figure 2). Signs of pure LMN dysfunction are considered as progressive muscular atrophy (PMA), whereas predominant UMN signs are associated with primary lateral sclerosis (PLS) [30]. PMA and PLS are both rare diseases and represent 5% of MND patients [27].

#### 2.3.1. UMN-Dominant ALS Variants

Patients can present predominant UMN dysfunction as in primary lateral sclerosis (PLS) or pseudobulbar palsy. The UMN predominant phenotype can then progress to ALS, which is observed in 40% of PLS cases [36]. Patients diagnosed with PLS for not meeting the diagnostic criteria for ALS can still slowly develop signs of LMN dysfunction and therefore present both UMN and LMN signs [27]. However, LMN involvement and limb atrophy in PLS is exceptionally rare [37] and the prognosis for PLS patients is better than that for patients diagnosed with ALS as symptom progression is relatively slow.

#### 2.3.2. LMN-Dominant ALS Variants

On the contrary, some patients can develop a LMN-dominant phenotype which can be defined as progressive muscular atrophy (PMA), and flail-arm or flail-leg syndrome variants. PMA patients are similar to classic ALS patients without obvious signs of UMN dysfunction. However 50% to 60% of PMA patients develop degeneration of upper motor neurons during the progression of the disease [38], and post-mortem histopathology has demonstrated that some PMA patients show UMN involvement which could not be detected upon clinical examination [39,40]. In patients with flail-arm or flail-leg syndromes, a LMN pattern of weakness and atrophy is observed in the upper limbs or lower limbs, respectively. Similar to PMA, flail-arm and flail-leg syndrome have been described as a LMN variant but can show UMN involvement in the later stages of disease [41]. Involvement of secondary sites should not occur within 12 months of initial onset [42] and prognosis for flail-arm and flail-leg syndrome is better than that seen in ALS, with median survival times of 5 to 6 years [41,43].

### 2.4. Non-Motor Involvement in ALS and Overlap with FTD

For many years, ALS was described as a neurodegenerative disorder with no extra-motor involvement. However, non-motor involvement is now accepted in the ALS phenotype [44], with neuroimaging demonstrating reduced grey matter in motor and non-motor brain regions of ALS patients [45], and histopathology suggesting widespread neuronal and glial TDP-43 pathology in the CNS [46]. In regards to symptomology, a low proportion of ALS patients experience non-motor impairment as a first indication of pathology (3% of sporadic cases and 15% of familial cases) [47]. It has been estimated that approximatively 35% of ALS patients present behavioural and/or cognitive changes (with 15% meeting the Neary criteria [48] for FTD diagnosis (ALS-FTD) [47]). The reported percentage seems to be much lower in most gene-specific studies and varies considerably between them, but it should be noted that the number of patients and studies for which these clinical parameters are reported is relatively small (Table S2). ALS and FTD are sometimes described as part of one continuum, with pure ALS patients (without any non-motor involvement) and pure FTD cases (for whom no motor dysfunction has been described) representing opposite ends of the spectrum.

ALS patients having FTD usually meet the criteria for behavioural variant FTD characterised by defects in cognitive functions, personality traits and behavioural collapse. Among ALS cases experiencing non-motor dysfunction, language (particularly deficits in verbal fluency) and cognition are the most affected categories [49], and apathy is the most frequently encountered personality impairment [47].

#### 2.4.1. Dementia in ALS Patients—ALS-FTD Variants

ALS-FTD diagnosis is made upon the presence of an ALS phenotype associated with behavioural or cognitive defects that fulfil FTD diagnostic criteria: (1) progressive impairment of behavioural/cognitive functions and observation of at least three behavioural symptoms defined by Rascvosky et al. [50]; or (2) loss of insight and/or presence of psychotic features associated with at least two Rascvosky et al. [50] symptoms; or (3) language impairment combined with semantic dementia (defined in [48]).

#### 2.4.2. Cognitive Changes in Non-Demented ALS Patients—ALSci and ALSbi Variants

Non-demented ALS patients presenting with behavioural impairment are classified as ALSbi-variant, while ALS patients experiencing cognitive impairment including language defects are considered to be ALSci variant [47]. Based on the revised diagnostic criteria from Strong et al. [51], ALS patients can be diagnosed as ALSci variant if either executive impairment (social cognition), or language dysfunction, or a combination of the two features are evident during diagnosis. Diagnostic criteria for ALSbi variant require apathy with or without other behavioural symptoms, or two or more behavioural changes, such as disinhibition, loss of sympathy/empathy, perseverative/stereotypic/compulsive behaviour, hyper orality/dietary change, loss of insight and psychotic symptoms.

## 3. Genetics of ALS

*Superoxide dismutase 1 (SOD1)* was the first gene demonstrated to be associated with ALS in 1993 [52]. *SOD1* is ubiquitously expressed in human cells and serves to protect them from harmful reactive oxygen species (ROS). Mutated forms of *SOD1* are believed to result in a toxic gain of function, provoking the presence of misfolded protein aggregates, increased endoplasmic reticulum (ER) stress, and oxidative stress and ultimately accelerating motor neuron degeneration [17].

In 2001, mutations in *ALSIN2 (ALS2)* were shown to be implicated in juvenile forms of ALS [53,54,55] and PLS [56]. The ALS2 protein has been found to act as a guanine nucleotide exchange factor for the GTPase, Rab5, which is in involved in endosome trafficking [57]. Mutations in *ALS2* have been shown to inhibit activation of Rab5 and its translocation to mitochondria, leaving *ALS2* mutated motor neurons more susceptible to oxidative stress [58]. However, in murine studies, genetic ablation of *ALS2* has failed to recapitulate the pathological features seen in ALS [59,60] although primary motor neurons from these mice did show greater sensitivity to oxidative stress and aberrant morphology, suggesting that *ALS2* mutations may indeed play a role in motor neuron susceptibility in ALS.

Genetic mutations were next reported in 2004 for the *senataxin (SETX)*, *angiogenin (ANG)*, and *vesicle-associated membrane protein-associated protein B (VAPB)* genes. *SETX* plays a role in numerous cellular functions including RNA metabolism and has been shown to regulate RNA polymerase II transcription termination [61] and its yeast homolog, SEN1, has been linked with processing of non-coding RNA [62]. *SETX* mutations are strongly associated with juvenile-onset ALS [63] and associations have been confirmed in American, Italian and Dutch cohorts [63,64,65]. ANG is highly expressed in the human central nervous system [66] and has been reported to show neuroprotective properties [67]. Indeed, expression of ALS-associated *ANG* variants has been shown to cause motor neuron death in cell culture models [67]. *ANG* has also been reported to play a role in the transcription of ribosomal RNA [68] and many ALS-associated variants are believed to elicit a loss of function in ANG, thus eliminating any neuroprotective functionality [69]. VAPB is a protein closely associated with the endoplasmic reticulum and is thought to be involved in the induction of the unfolded protein response (UPR) [70], as well as cellular processes including lipid transport [71], protein secretion [72], and calcium homeostasis [73]. The P56S mutation in *VAPB* has been implicated in an early-onset and slow-progressing form of fALS [74] and follow-up studies have highlighted how this mutation can result in nuclear envelope defects [75], and provoke VAPB ER aggregates [72]. However, murine models expressing the P56S mutation show widespread VAPB aggregates but demonstrate no motor neuron pathology or ALS phenotypes [76].

The next genetic mutation associated with ALS did not arrive until 2008, when mutations in *TAR DNA-binding protein (TARDBP)*, encoding TDP-43, were reported in patients [77]. TDP-43 is a RNA/DNA-binding protein that plays important roles in several RNA metabolism processes [78]. Ubiquitinated TDP-43 was first shown to be present in CNS inclusions of ALS patients in 2006 [79] and subsequent studies have confirmed TDP-43 as the major protein component of pathological inclusions present in approximately 90% of ALS patients [80]. However, TDP-43 pathology is not unique to ALS and has been reported in numerous neurodegenerative conditions including FTD [79], Parkinson’s disease [81], Huntington’s disease [82], Alzheimer’s disease [83], and dementia with Lewy bodies [84].

Then, in 2009, multiple mutations in the nuclear RNA-binding protein, *Fused in Sarcoma (FUS)* and *FIG4 phosphoinositide 5-phosphatase (FIG4)*, were associated with ALS [85,86]. FUS is another RNA/DNA-binding protein involved in mechanisms of RNA splicing and DNA repair [87] and is implicated in both ALS and FTD [88]. Mutations in *FUS*, particularly those near the nuclear localisation signal (NLS) domain, cause cytoplasmic protein mislocalisation and are associated with a severe phenotype in murine models [89]. *FIG4* is involved in vesicle trafficking due to its role in the regulation of the membrane bound phosphoinositide, PI(3,5)P2 [90]. Mutations in *FIG4* were initially shown to cause neurodegeneration in Charcot–Marie–Tooth (CMT) neuropathy [91]. However, others have questioned the role of *FIG4* in ALS pathology after failing to find pathogenic mutations in their Taiwanese [92] and Italian [93] cohorts.

In 2010, mutations in *Optineurin (OPTN)*, *Spatacsin paraplegia 11 (SPG11)*, *Valosin-containing protein (VCP)*, and *Ataxin-2 (ATXN2)* were all implicated in ALS. Three different *OPTN* mutations were identified in ALS patients [94] and researchers were able to demonstrate the increased immunoreactivity of OPTN in both TDP-43 and SOD1 inclusions found in the spinal cord of sALS patients, suggesting a role for *OPTN* in general ALS pathogenesis.

The link between *SPG11* and ALS was established when mutations were found to be associated with autosomal recessive juvenile ALS [95]. Mutations to *SPG11* are the most common cause of autosomal recessive hereditary spastic paraplegia [96] and loss of function mutations have been shown to elicit lysosomal dysfunction and UMN + LMN degeneration in mice [97]. *ATXN2* encoding the ataxin-2 polyglutamine (polyQ) protein was associated with ALS when researchers identified the presence of intermediate length polyQ expansions (27-33 Qs) in 4.7% of their North-American ALS cohort [98]. Ataxin-2 protein has been shown to regulate mRNA stability and translation [99,100] and upregulation of the fly homolog of Ataxin-2 was found to enhance neurodegeneration in *Drosophila* via its interaction with wild-type and mutated forms of TDP-43 [98]. Involvement of *Ataxin-2* in ALS pathogenesis has since been confirmed in European and Chinese patient cohorts [101,102]. VCP is an ATP-driven chaperone protein that plays a role in ubiquitin-regulated protein degradation [103], autophagy [104], and mRNA processing [105,106]. *VCP* mutations were shown to be present in 1–2% of familial ALS patients in an Italian cohort [107] and mice expressing ALS-associated *VCP* mutations have been shown to develop a slow-progressing ALS phenotype [108].

In 2011, mutations in *ubiquilin-2 (UBQLN2)*, *sequestosome-1 (SQSTM1)*, and *chromosome 9 open reading frame 72 (C9orf72)* were discovered. Ubiquilin-positive inclusions have been implicated in both sALS and fALS [109], whilst mutations in *SQSTM1* have been observed in rare ALS and FTD cases [110] and can be shown to lead to p62 protein inclusions in motor neurons of both patient groups [111]. The G4C2 hexanucleotide repeat expansion mutation (HREM) within *C9orf72* [112,113] is perhaps the most significant genetic mutation associated with ALS thus far, and is estimated to be present in 34% of familial cases, and 5% of sporadic cases in Europe [19,114]. In healthy subjects, the G4C2 repeat length ranges from 2 to 23 units [112], whilst intermediate expansions ranging from 24 to 30 [115] and large expansions ranging from 30 to many hundreds of units have been observed in ALS patients [112,116]. Although rare, *C9orf72* expansions have been implicated in other neurodegenerative and psychiatric diseases including PD [117] and Schizophrenia [118], suggesting a wider role for *C9orf72* in neuropathology and perhaps offering some insight towards the heterogeneous phenotype seen in *C9orf72* ALS.

In 2012, *Profilin 1 (PFN1)* was implicated in familial and sporadic cases of ALS [119]. Mutant *PFN1* has been shown to cause motor neuron degeneration through the formation of insoluble aggregates and disrupted cytoskeleton dynamics in mice [120] and co-aggregation of PFN1 and TDP-43 has been reported in cell lines expressing mutant *PFN1* [119].

Then, in 2013, *heterogeneous nuclear ribonucleoprotein A1 (hnRNPA1)* was reported to be involved in ALS after researchers identified three *hnRNPA1* variants—two of which were associated with familial ALS and the other of which was associated with a sporadic case [121]. hnRNPA1 is known to colocalise with TDP-43 [121] and post-mortem studies have shown that motor neurons of ALS patients display marked reductions in hnRNPA1 alongside concomitant TDP-43 inclusions [122].

In 2014, mutations in *Tubulin alpha-4A (TUBA4A)* and *Matrin-3 (MATR3)* were implicated in ALS. Mutations in *TUBA4A* were first identified in a European and American cohort [123] and then validated in Belgian and Chinese cohorts in 2017 and 2018 [124,125]. *TUBA4A* mutations have been shown to cause cytoskeletal defects in primary motor neurons [123] and are recognised as a rare cause of ALS and FTD [125].

*MATR3* was first associated with ALS after exome sequencing identified mutations in Italian, UK and US kindreds, alongside increased levels of MATR3 protein in spinal cord sections of ALS patients relative to controls [126]. MATR3 has been found to interact with TDP-43 and both proteins were shown to co-aggregate in skeletal muscle tissue of ALS patients [126]. *MATR3* is known to play various roles in RNA metabolism and alternative splicing [127,128] and recent evidence suggests ALS-associated *MATR3* mutations play a role in defective nuclear export of FUS and TDP-43 mRNA [129]

In 2015, *NIMA-related kinase 1 (NEK1)* was recognised as an ALS-risk gene [130] and was shown to interact with two other ALS genes, *ALS2* and *VAPB*—both of which are involved in endosomal trafficking. Subsequent studies provided further evidence for the pathogenic role of *NEK1* in ALS [131,132] and pathway analyses have shown NEK1 to interact with C21orf72—both of which are involved in DNA repair mechanisms [133]. Mutations in *Tank-binding kinase 1 (TBK1)* were also associated with ALS in 2015 after exome sequencing identified eight loss of function mutations in 13 fALS pedigrees [134].

*Cyclin F (CCNF)* was implicated in ALS in 2016 with variants identified in both familial and sporadic cases [135]. In the same study, researchers were able to demonstrate how mutant *CCNF* led to aberrant ubiquitination and aggregation of proteins including TDP-43. More recently, CCNF was shown to be a binding partner of another ALS protein, VCP. Binding of mutated CCNF to VCP increased VCP ATPase activity, which in turn led to increased TDP-43 aggregation in U20S cells [136].

Then, in 2018, the most recent genetic mutations implicated in ALS were discovered when research demonstrated the pathological involvement of *Kinesin family member 5A (KIF5A)* [137]. KIF5A is a protein expressed specifically in neurons and is involved in regulating neuronal microtubule dynamics [138,139]. *KIF5A* is also associated with spastic paraplegia and Charcot–Marie–Tooth neuropathy [140] and mutations have been reported in ALS patients in Chinese [141], European [142,143], and US cohorts [137].

## 4. Correlation of Genotype/Phenotype: Methods, Results and Discussion

To evaluate whether there is a correlation between associated genes and phenotype in ALS, a systematic search of original papers was performed using key words summarised in Table S1, while adhering to PRISMA guidelines (see checklist in Supplementary Materials).

### 4.1. Protocol

A systematic search was performed in PubMed using the key words: ALS, genotype phenotype, patient, and onset. To make sure that clinical data would also be obtained for rare genes involved in ALS and listed in Vijayakumar et al. [14], the following search terms were added: ALS, phenotype, patient and the gene name such as *TBK1*, *VCP*, *SQSTM1*, *CCNF*, *NEK1*, *OPTN*, *FIG4*, *PFN1*, *ATXN2*, *VAPB*, *ANG*, *ALS2*, *SPG11*, *UBQLN2*, *KIF5A*, and *MATR3*. There were no language, type of study, or publication date restrictions.

### 4.2. Eligibility Criteria

**Figure 3:**
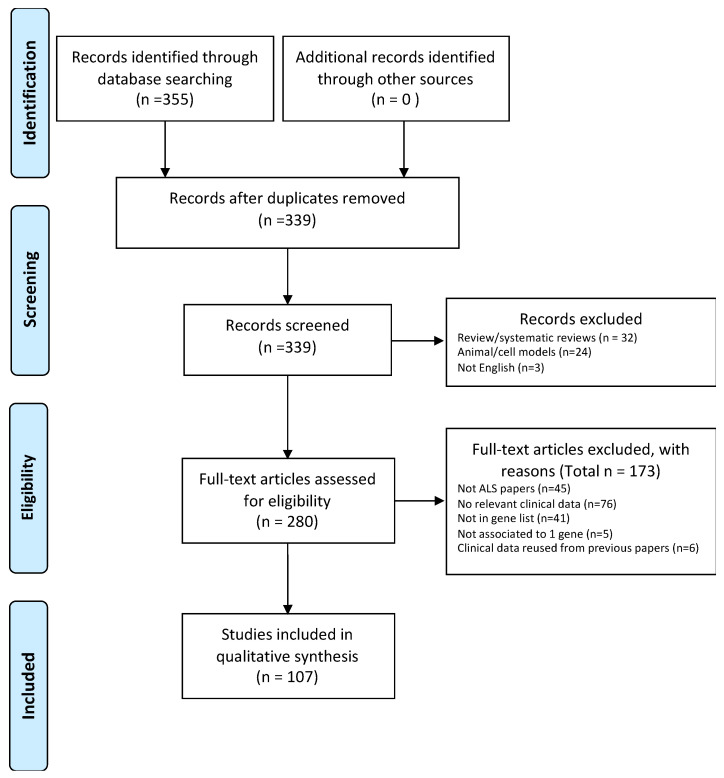
PRISMA flow chart showing how studies have been selected.

The search combining the different key words resulted in 355 articles. Reviews and duplicated papers were excluded. To avoid redundancy, papers re-using previously published clinical data were excluded. All studies used in the systematic review were peer-reviewed, written in English, and published original clinical data related to patients affected by monogenic forms of ALS. At least one of the following parameters had to be described in the paper: age of onset, site of onset, motor neuron population being affected (UMN, LMN, UMN+LMN), disease duration, number of patients with FTD, and number of patients with cognitive impairment. A total of 107 papers were then eligible for the analysis (see PRISMA flow chart in Figure 3).

### 4.3. Data Extractions and Synthesis

The papers were thoroughly reviewed by OC, LLG, VM and SD. Key information was extracted from each study, and grouped into cohort characteristics (ethnicity/ country of the study, number of patients), age of onset (distribution and mean and standard deviation (SD)), site of onset (spinal, bulbar, respiratory, other/unknown), motor neurons being affected (UMN, LMN, UMN+LMN), disease duration (mean and SD), percentage of patients with FTD, and percentage of patients with cognitive impairment. All data are collated per gene in Table S2.

**Figure 4:**
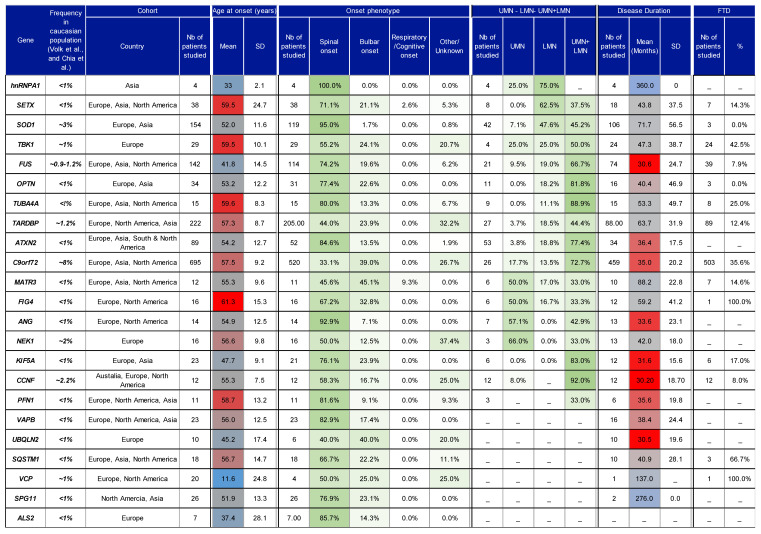
Table summarising the phenotypes observed in ALS patients with different mutations. A detailed version of this table is accessible as supplemental data (Table S2). PubMed was searched to identify published studies reporting genotype–phenotype data for 23 genes. Column 2 indicates the frequency of genes observed in Caucasian populations described in previous reviews (Volk et al. [18] and Chia et al. [167]). The ethnicity/origin of cohorts reported across studies is specified in Table S2 and summarised in column 3. For each parameter reported in this table, the number of patients is given in the first subcolumn for each category. For age of onset, motor neuron impairments, disease duration, and FTD, the weighted mean ± SD, and weighted percentages are given, taking into account the numbers of patients studied. Data for each gene were collected from the following reference studies, then summarized: hnRNPA1: [168]; SETX: [169,170,171,172,173]; SOD1: [24,92,160,161,171,172,174,175,176,177,178,179,180,181,182,183,184,185,186,187,188]; TBK1: [189,190,191,192]; FUS: [24,92,145,146,148,170,171,172,179,180,187,188,193,194,195,196]; OPTN: [171,180,187,197,198,199,200,201,202,203]; TUBA4A: [123,188,204,205]; TARDBP: [171,172,179,180,187,188,192,206,207,208,209,210,211]; ATXN2: [101,102,171,180,188,212,213,214]; C9orf72: [114,170,171,175,180,188,206,215,216,217,218,219,220,221,222,223,224,225,226]; MATR3: [227,228,229,230,231]; FIG4: [171,192,232]; ANG: [171,180,187,233,234,235]; NEK1: [131,236]; KIF5A: [137,141,143,237]; CCNF: [135]; PFN1: [238,239,240,241]; VAPB: [171,242,243]; UBQLN2: [244,245]; SQSTM1: [171,175,192,246]; VCP: [170,171,175,192,247]; SPG11: [179,248]; ALS2: [55,170]. Results from each separate study are shown in Table S2. Gradient colour for age of onset from dark blue to dark red: dark blue, 16 years; dark red, 60 years. Gradient colour for site of onset and for motor neuron impairment distributions: white, 0%; green, 100%.

For the summary Table (Figure 4), the age of onset and disease duration are presented as the weighted mean ± SD, and the site of onset, motor neuron impairments, and FTD comorbidity are presented as weighted percentages, in all cases taking into account the number of patients studied as described below:

Mean = mean of the parameter of interest given in the referenced study;

n = number of patients studied for the corresponding parameter in the given study;

*Sx*^2^ = *SD*^2^(*n*−1) + ((*Sx*)^2^/*n*)

$\mathrm{Weighted}\;\mathrm{mean}=\frac{\sum \mathrm{Sx}}{\sum n}$

$\mathrm{Weighted}\;\mathrm{SD}=\sqrt[{2}]{\frac{(\sum \left({\mathrm{Sx}}^{2}\right)-{\left(\sum \mathrm{Sx}\right)}^{2})/\sum n}{(\sum n)-1}}$

### 4.4. Characteristics of Studies

A total of 1630 ALS patients were included in the systematic review. The total number of reported patients for each gene is shown in Figure 4 column 4. As not all studies reported all clinical parameters, the total number of patients studied for each parameter is reported in the first subcolumn for each parameter. On average, 59% of the population was male, with considerable variation between genes (See Table S2). Most of the studies were conducted in Europe, North America and Asia.

### 4.5. Overall Findings and Discussion

For most genetic forms of ALS reported in Table S2 and in Figure 4, the age of onset ranges between 50 and 70 years old. Exceptions to this include cases of juvenile ALS, which are observed with mutations in *SPG11* [95,144], *FUS* [145,146] and *ALS2* [53,55] (Table S2). Whilst *FUS* patients are known to show considerable variation in phenotype, with some showing early onset and fast progression, others show a later age of onset and a slower-progressing phenotype [147]. This variation in the *FUS* phenotype has been hypothesised to arise due to the different effects exerted by missense and truncating mutations [148]. Interestingly, the studies reviewed here suggest that *FUS* mutations are indeed associated with a relatively early age of onset (41.8 ± 14.5 years) and a fast-progressing phenotype, with average disease duration lasting 30.6 months (Figure 4). Another gene sometimes associated with early-onset ALS is *SETX*. Patients with *SETX* mutations have been reported to display a slow-progressing phenotype in which bulbar and respiratory muscles seem largely unaffected [149]. However, in one reported case, a patient did go on to experience bulbar symptoms 3 years after onset [150]. Moreover, from the studies retrieved in this review, *SETX* patients do not show an early age of onset nor a particularly slow phenotype. For instance, the average age of onset for *SETX* patients was 59.5 ± 24.7 years with an average disease duration of 43.8 ± 37.5 months.

Many ALS-associated genes show variation in site of onset. Among the 22 genes included in Figure 4, cases of spinal onset are predominant in 19. This is in line with previous findings that suggest spinal onset accounts for approximately two-thirds of ALS cases [32]. For example, *SOD1*, *hnRNAP1*, *TUBA4A*, and *ALS2* show a high percentage of patients with spinal onset (>80%), while spinal onset in *VCP*, *NEK1*, and *TBK1* cases accounted for 50%, 50% and 55% of cases, respectively. Some other ALS-associated gene mutations were associated with a lower proportion of spinal onset, e.g., 33% of *C9orf72* cases, and 40% of *UBQLN2* cases. However, previous research suggests that *C9orf72* ALS demonstrates frequent occurrence of both spinal [151] and bulbar onset [152]. Moreover, it has been reported that site of onset in *C9orf72* ALS can be used to predict disease duration. For instance, the average age of onset in patients with spinal onset was 59.3 years, increasing to 62.3 years in patients with bulbar onset, and male patients with spinal onset seem to display a faster-progressing phenotype [153].

A striking 95% of *SOD1* cases were classified as spinal onset. Indeed, animal studies have provided support for the notion that *SOD1* pathology begins at the periphery and proceeds in a retrograde manner [154,155]. Recently, a homozygous mutation that eliminates the enzymatic activity of *SOD1* was found to result in a severe LMN phenotype and mild cerebellar atrophy in a young child [156] and the presence of a *SOD1* p.D12Y variant was shown to result in a LMN-predominant phenotype [157]. Similarly, seven studies reported a non-negligible percentage of patients with pure LMN signs (Table S2, Figure 4, 47.6% pure LMN vs. 45.2% UMN+LMN, [158,159,160,161]). Overall, these studies seem to suggest that *SOD1* mutations exert profound effects at the distal nerve. In addition, the observation that both overexpression, and absence of *SOD1* activity lead to pathology should be an important consideration in the development of therapeutics that aim to alter *SOD1* levels as a novel treatment in ALS [162].

Figure 4 was sorted in descending order for the percentage of patients showing LMN signs. Not all studies reported UMN and/or LMN signs, and thus the percentage given in this table only represents a small proportion of the studies (see Table S2 for more details). However, it is interesting to see that the majority of the gene mutations do indeed elicit a phenotype that is characterised by both UMN+LMN signs, consistent with the classical clinical definition of ALS. *FUS*, *C9orf72* and *TARDBP* all demonstrated increased presence of both UMN and LMN signs with both neuronal populations affected in 66.7%, 72.7% and 44.4%, respectively. Surprisingly, only 33% of *FIG4*, *PFN1*, *MATR3* and *NEK1* cases showed both UMN and LMN signs, although it should be noted that 4 of the 14 studies reviewed in relation to these genes did not provide details regarding the pattern of motor neuron involvement. Some ALS-associated genes demonstrated >20% of patients with pure LMN signs (*SOD1*, *FUS*, *PFN1*, *ATXN2*, *TARDBP*, *TBK1*, and *hnRNPA1*), while pure UMN signs had >20% preponderance in several genes (*ANG*, *TBK1*, *FIG4*, *MATR3*, *NEK1*, *hnRNPA1*).

Finally, the current review also aimed to collect information regarding the prevalence of cognitive impairments and FTD in ALS. FTD was most frequent in cases with mutations in either *C9orf72*, *SQSTM1*, or *TBK1* (36%, 67%, and 43%, respectively, Figure 4). Indeed, *C9orf72* [163], *SQSTM1* [164], and *TBK1* [165] have all previously been linked with FTD onset. However, in a large screen of 121 patients with FTD, genetic mutations were successfully identified only in *C9orf72* and *SQSTM1*, whilst no *TBK1* variants were identified [166]. It is also worth noting that despite the frequent association between *TARDBP* and FTD, only 12% of cases reviewed here were found to have concomitant FTD symptoms. In relation to general cognitive functioning, reports of impairment were observed across 10 ALS-associated genes, although the number of patients studied for this parameter were often quite low (Table S2), rendering it difficult to form conclusions.

## 5. Conclusions

Over 150 years have passed since ALS was first reported by Charcot and still the aetiology of the disease remains elusive. Although research is progressing and genetic studies continue to identify novel gene associations [14,249,250,251], many questions remain surrounding the pathological mechanisms associated with already established mutations, their role in the ALS phenotype, and the as yet undiscovered mechanisms that underlie sporadic onset of disease. Here, we have performed a systematic review in an attempt to highlight genotype–phenotype correlations for 23 of the more commonly reported mutated genes in ALS. This has proven to be challenging as many genetic studies do not capture or report a complete summary of clinical data. Whilst it is understandable that such data are difficult to acquire, we hope to illustrate that there is a need for improved and more widely available clinical and informatics resources that would enable genotype–phenotype associations to be easily visualised in ALS.

Whilst we have illustrated the relationship between commonly reported mutated genes and various clinical measures including age and site of onset, disease duration and motor neuron involvement, a limitation of the current review is that we do not consider variation among phenotypes of patients having different mutations of the same gene. For many genes involved in ALS, including *FUS*, *SOD1*, and *TARDBP*, the phenotype may be different depending upon the specific genetic mutation in question. In *SOD1* patients, for instance, the A4V mutation results in a much more aggressive phenotype (death occurring ~1.2 years after onset [252]) than the H46R mutation, for which patients show a relatively mild phenotype (duration of ~17 years [253]). It could be of value in future work to comprehensively review variations in genotype–phenotype correlations among the different mutations reported by single-gene studies, which in turn could contribute towards a comprehensive database of ALS genotype–phenotype correlation. Such a resource could ultimately improve our mechanistic understanding of ALS by enabling a more robust assessment of how the ALS phenotype responds to different variants across multiple genes.

Additional limitations include that many of the studies surveyed are relatively small, involving low numbers of patients, and that, as well as only a subset of studies reporting clinical breakdown of phenotype, ethnic breakdown is also not always reported and some ethnicities have minimal representation.

Despite these limitations, the collected data reveal a landscape of highly variable phenotypic associations, underlining the complexity of the disease, and the need for nuanced approaches to the development of clinical assays and therapeutics.

## Figures and Tables

**Table 1:** ALS age of onset variability and their clinical features. Summary of clinical features for ALS in different age periods from Chio et al. [28], Forbes et al. [12], Swinnen et al. [27], Turner at al [26], Sabetelli et al. [29], and Kiernan et al. [25]. In addition to the classical ALS phenotype with age of onset ranging from 45 to 70 years old (mean age ~ 61 years old), three additional age of onset periods (columns) have been observed. Male to female ratios, genetic characteristics, site of onset, estimated survival time, and clinical features are shown where applicable. sALS: sporadic ALS. fALS: familial ALS. MN: motor neurons. UMN: upper motor neurons. LMN: lower motor neurons. -: no data.

	Juvenile	Young	Mid–Late Adulthood	Elderly
Age of onset	≤25 years old	25 to 45 years old	45 to 70 years old	>70 years old
M:F ratio	_	3–3.6:1	1.3–1.56:1	1:1.25
Genetics	Mostly familial cases (*FUS*, *SETX*, *ALS2* mutations)	Mostly familial	~90% sALS ~10% fALS	_
Site of onset:				
Limb onset	_	_	~70%	~40%
Bulbar onset	_	~16%	~25%	~50% (M:F = 1:1.6)
Respiratory/cognitive onset	_	_	~5%	_
Survival (from symptoms onset)	Generally longer survival > 10 years		Variable: 50%: <30 months 5–10%: 5–10 years	~20 months
MN involvement:				
UMN+LMN	_	~40%	~80%	~72%
UMN-predominant	Predominant	~60%	~17%	_
LMN-predominant	_	_	_	~19%

## Supplementary Materials

The following are available online at https://www.mdpi.com/2075-4426/10/3/58/s1, **Table S1**: Key words used in PubMed literature search. **Table S2:** Collated data from clinical studies on monogenic forms of ALS. **Table S3:** PRISMA checklist for systematic review.

## References

[1] Logroscino G., Piccininni M., Marin B., Nichols E., Abd-Allah F., Abdelalim A., Alahdab F., Asgedom S.W., Awasthi A., Chaiah Y. (2018). Global, regional, and national burden of motor neuron diseases 1990–2016: A systematic analysis for the Global Burden of Disease Study 2016. Lancet Neurol..

[2] Gordon P.H. (2013). Amyotrophic Lateral Sclerosis: An update for 2013 Clinical Features, Pathophysiology, Management and Therapeutic Trials. Aging Dis..

[3] Talbott E.O., Malek A.M., Lacomis D. (2016). The epidemiology of amyotrophic lateral sclerosis. Handbook of Clinical Neurology.

[4] Chiò A., Logroscino G., Traynor B.J., Collins J., Simeone J.C., Goldstein L.A., White L.A. (2013). Global Epidemiology of Amyotrophic Lateral Sclerosis: A Systematic Review of the Published Literature. Neuroepidemiology.

[5] Mehta P., Kaye W., Raymond J., Wu R., Larson T., Punjani R., Heller D., Cohen J., Peters T., Muravov O. (2014). Prevalence of Amyotrophic Lateral Sclerosis—United States. Morb. Mortal. Wkly. Rep..

[6] Arthur K.C., Calvo A., Price T.R., Geiger J.T., Chiò A., Traynor B.J. (2016). Projected increase in amyotrophic lateral sclerosis from 2015 to 2040. Nat. Commun..

[7] Salameh J., Brown R., Berry J. (2015). Amyotrophic Lateral Sclerosis: Review. Semin. Neurol..

[8] Brooks B.R., Antel J., Bradley W., Cardy P., Carpenter S., Chou S., Conradi S., Daube J., Denys E.H., Festoff B. (1994). El Escorial World Federation of Neurology criteria for the diagnosis of amyotrophic lateral sclerosis. J. Neurol. Sci..

[9] Brooks B.R., Miller R.G., Swash M., Munsat T.L. (2000). El Escorial revisited: Revised criteria for the diagnosis of amyotrophic lateral sclerosis. Amyotroph. Lateral Scler. Other Motor Neuron Disord..

[10] de Carvalho M., Dengler R., Eisen A., England J.D., Kaji R., Kimura J., Mills K., Mitsumoto H., Nodera H., Shefner J. (2008). Electrodiagnostic criteria for diagnosis of ALS. Clin. Neurophysiol..

[11] Chiò A., Moglia C., Canosa A., Manera U., Vasta R., Brunetti M., Barberis M., Corrado L., D’Alfonso S., Bersano E. (2019). Cognitive impairment across ALS clinical stages in a population-based cohort. Neurology.

[12] Forbes R.B., Colville S., Swingler R.J., Scottish ALS/MND Register (2004). The epidemiology of amyotrophic lateral sclerosis (ALS/MND) in people aged 80 or over. Age Ageing.

[13] Al-Chalabi A., Hardiman O., Kiernan M.C., Chiò A., Rix-Brooks B., van den Berg L.H. (2016). Amyotrophic lateral sclerosis: Moving towards a new classification system. Lancet Neurol..

[14] Vijayakumar U.G., Milla V., Cynthia Stafford M.Y., Bjourson A.J., Duddy W., Duguez S.M.-R. (2019). A Systematic Review of Suggested Molecular Strata, Biomarkers and Their Tissue Sources in ALS. Front. Neurol..

[15] Wingo T.S., Cutler D.J., Yarab N., Kelly C.M., Glass J.D. (2011). The Heritability of Amyotrophic Lateral Sclerosis in a Clinically Ascertained United States Research Registry. PLoS ONE.

[16] Al-Chalabi A., Fang F., Hanby M.F., Leigh P.N., Shaw C.E., Ye W., Rijsdijk F. (2010). An estimate of amyotrophic lateral sclerosis heritability using twin data. J. Neurol. Neurosurg. Psychiatry.

[17] Mathis S., Goizet C., Soulages A., Vallat J.-M., Masson G. (2019). Le Genetics of amyotrophic lateral sclerosis: A review. J. Neurol. Sci..

[18] Volk A.E., Weishaupt J.H., Andersen P.M., Ludolph A.C., Kubisch C. (2018). Current knowledge and recent insights into the genetic basis of amyotrophic lateral sclerosis. medizinische Genet..

[19] Zou Z.-Y., Zhou Z.-R., Che C.-H., Liu C.-Y., He R.-L., Huang H.-P. (2017). Genetic epidemiology of amyotrophic lateral sclerosis: A systematic review and meta-analysis. J. Neurol. Neurosurg. Psychiatry.

[20] Yoshida M., Takahashi Y., Koike A., Fukuda Y., Goto J., Tsuji S. (2010). A mutation database for amyotrophic lateral sclerosis. Hum. Mutat..

[21] McCann E.P., Williams K.L., Fifita J.A., Tarr I.S., O’Connor J., Rowe D.B., Nicholson G.A., Blair I.P. (2017). The genotype-phenotype landscape of familial amyotrophic lateral sclerosis in Australia. Clin. Genet..

[22] Li H.-F., Wu Z.-Y. (2016). Genotype-phenotype correlations of amyotrophic lateral sclerosis. Transl. Neurodegener..

[23] Sabatelli M., Conte A., Zollino M. (2013). Clinical and genetic heterogeneity of amyotrophic lateral sclerosis. Clin. Genet..

[24] Millecamps S., Salachas F., Cazeneuve C., Gordon P., Bricka B., Camuzat A., Guillot-Noël L., Russaouen O., Bruneteau G., Pradat P.-F. (2010). SOD1, ANG, VAPB, TARDBP, and FUS mutations in familial amyotrophic lateral sclerosis: Genotype-phenotype correlations. J. Med. Genet..

[25] Kiernan M.C., Vucic S., Cheah B.C., Turner M.R., Eisen A., Hardiman O., Burrell J.R., Zoing M.C. (2011). Amyotrophic lateral sclerosis. Lancet.

[26] Turner M.R., Barnwell J., Al-Chalabi A., Eisen A. (2012). Young-onset amyotrophic lateral sclerosis: Historical and other observations. Brain.

[27] Swinnen B., Robberecht W. (2014). The phenotypic variability of amyotrophic lateral sclerosis. Nat. Rev. Neurol..

[28] Chiò A., Logroscino G., Hardiman O., Swingler R., Mitchell D., Beghi E., Traynor B.G. (2009). On Behalf of the Eurals Consortium Prognostic factors in ALS: A critical review. Amyotroph. Lateral Scler..

[29] Sabatelli M., Madia F., Conte A., Luigetti M., Zollino M., Mancuso I., Lo Monaco M., Lippi G., Tonali P. (2008). Natural history of young-adult amyotrophic lateral sclerosis. Neurology.

[30] Tard C., Defebvre L., Moreau C., Devos D., Danel-Brunaud V. (2017). Clinical features of amyotrophic lateral sclerosis and their prognostic value. Rev. Neurol..

[31] Garden G.A., La Spada A.R. (2012). Intercellular (mis)communication in neurodegenerative disease. Neuron.

[32] Hardiman O., Al-Chalabi A., Chio A., Corr E.M., Logroscino G., Robberecht W., Shaw P.J., Simmons Z., van den Berg L.H. (2017). Amyotrophic lateral sclerosis. Nat. Rev. Dis. Prim..

[33] Bäumer D., Talbot K., Turner M.R. (2014). Advances in motor neurone disease. J. R. Soc. Med..

[34] van Es M.A., Hardiman O., Chio A., Al-Chalabi A., Pasterkamp R.J., Veldink J.H., van den Berg L.H. (2017). Amyotrophic lateral sclerosis. Lancet.

[35] Lillo P., Hodges J.R. (2009). Frontotemporal dementia and motor neurone disease: Overlapping clinic-pathological disorders. J. Clin. Neurosci..

[36] D’Amico E., Pasmantier M., Lee Y.-W., Weimer L., Mitsumoto H. (2013). Clinical evolution of pure upper motor neuron disease/dysfunction (PUMMD). Muscle Nerve.

[37] Statland J.M., Barohn R.J., McVey A.L., Katz J.S., Dimachkie M.M. (2015). Patterns of Weakness, Classification of Motor Neuron Disease, and Clinical Diagnosis of Sporadic Amyotrophic Lateral Sclerosis. Neurol. Clin..

[38] Rowland L.P. (2010). Progressive muscular atrophy and other lower motor neuron syndromes of adults. Muscle Nerve.

[39] Riku Y., Atsuta N., Yoshida M., Tatsumi S., Iwasaki Y., Mimuro M., Watanabe H., Ito M., Senda J., Nakamura R. (2014). Differential motor neuron involvement in progressive muscular atrophy: A comparative study with amyotrophic lateral sclerosis. BMJ Open.

[40] Liewluck T., Saperstein D.S. (2015). Progressive Muscular Atrophy. Neurol. Clin..

[41] Wijesekera L.C., Mathers S., Talman P., Galtrey C., Parkinson M.H., Ganesalingam J., Willey E., Ampong M.A., Ellis C.M., Shaw C.E. (2009). Natural history and clinical features of the flail arm and flail leg ALS variants. Neurology.

[42] Zou Z.-Y., Chen S.-D., Feng S.-Y., Liu C.-Y., Cui M., Chen S., Feng S.-M., Dong Q., Huang H., Yu J.-T. (2020). Familial flail leg ALS caused by PFN1 mutation. J. Neurol. Neurosurg. Psychiatry.

[43] Garg N., Park S.B., Vucic S., Yiannikas C., Spies J., Howells J., Huynh W., Matamala J.M., Krishnan A.V., Pollard J.D. (2017). Differentiating lower motor neuron syndromes. J. Neurol. Neurosurg. Psychiatry.

[44] Goldstein L.H., Abrahams S. (2013). Changes in cognition and behaviour in amyotrophic lateral sclerosis: Nature of impairment and implications for assessment. Lancet Neurol..

[45] Shen D., Cui L., Fang J., Cui B., Li D., Tai H. (2016). Voxel-Wise Meta-Analysis of Gray Matter Changes in Amyotrophic Lateral Sclerosis. Front. Aging Neurosci..

[46] Geser F., Brandmeir N.J., Kwong L.K., Martinez-Lage M., Elman L., McCluskey L., Xie S.X., Lee V.M.-Y., Trojanowski J.Q. (2008). Evidence of Multisystem Disorder in Whole-Brain Map of Pathological TDP-43 in Amyotrophic Lateral Sclerosis. Arch. Neurol..

[47] Crockford C., Newton J., Lonergan K., Chiwera T., Booth T., Chandran S., Colville S., Heverin M., Mays I., Pal S. (2018). ALS-specific cognitive and behavior changes associated with advancing disease stage in ALS. Neurology.

[48] Neary D., Snowden J.S., Gustafson L., Passant U., Stuss D., Black S., Freedman M., Kertesz A., Robert P.H., Albert M. (1998). Frontotemporal lobar degeneration: A consensus on clinical diagnostic criteria. Neurology.

[49] Bak T.H., Chandran S. (2012). What wires together dies together: Verbs, actions and neurodegeneration in motor neuron disease. Cortex.

[50] Rascovsky K., Hodges J.R., Knopman D., Mendez M.F., Kramer J.H., Neuhaus J., van Swieten J.C., Seelaar H., Dopper E.G.P., Onyike C.U. (2011). Sensitivity of revised diagnostic criteria for the behavioural variant of frontotemporal dementia. Brain.

[51] Strong M.J., Abrahams S., Goldstein L.H., Woolley S., Mclaughlin P., Snowden J., Mioshi E., Roberts-South A., Benatar M., HortobáGyi T. (2017). Amyotrophic lateral sclerosis—Frontotemporal spectrum disorder (ALS-FTSD): Revised diagnostic criteria. Amyotroph. Lateral Scler. Front. Degener..

[52] Rosen D.R., Siddique T., Patterson D., Figlewicz D.A., Sapp P., Hentati A., Donaldson D., Goto J., O’Regan J.P., Deng H.X. (1993). Mutations in Cu/Zn superoxide dismutase gene are associated with familial amyotrophic lateral sclerosis. Nature.

[53] Yang Y., Hentati A., Deng H.-X., Dabbagh O., Sasaki T., Hirano M., Hung W.-Y., Ouahchi K., Yan J., Azim A.C. (2001). The gene encoding alsin, a protein with three guanine-nucleotide exchange factor domains, is mutated in a form of recessive amyotrophic lateral sclerosis. Nat. Genet..

[54] Hadano S., Hand C.K., Osuga H., Yanagisawa Y., Otomo A., Devon R.S., Miyamoto N., Showguchi-Miyata J., Okada Y., Singaraja R. (2001). A gene encoding a putative GTPase regulator is mutated in familial amyotrophic lateral sclerosis 2. Nat. Genet..

[55] Sheerin U.-M., Schneider S.A., Carr L., Deuschl G., Hopfner F., Stamelou M., Wood N.W., Bhatia K.P. (2014). ALS2 mutations: Juvenile amyotrophic lateral sclerosis and generalized dystonia. Neurology.

[56] Eymard-Pierre E., Lesca G., Dollet S., Santorelli F.M., di Capua M., Bertini E., Boespflug-Tanguy O. (2002). Infantile-Onset Ascending Hereditary Spastic Paralysis Is Associated with Mutations in the Alsin Gene. Am. J. Hum. Genet..

[57] Otomo A. (2003). ALS2, a novel guanine nucleotide exchange factor for the small GTPase Rab5, is implicated in endosomal dynamics. Hum. Mol. Genet..

[58] Hsu F., Spannl S., Ferguson C., Hyman A.A., Parton R.G., Zerial M. (2018). Rab5 and Alsin regulate stress-activated cytoprotective signaling on mitochondria. Elife.

[59] Devon R.S., Orban P.C., Gerrow K., Barbieri M.A., Schwab C., Cao L.P., Helm J.R., Bissada N., Cruz-Aguado R., Davidson T.-L. (2006). Als2-deficient mice exhibit disturbances in endosome trafficking associated with motor behavioral abnormalities. Proc. Natl. Acad. Sci. USA.

[60] Cai H. (2005). Loss of ALS2 Function Is Insufficient to Trigger Motor Neuron Degeneration in Knock-Out Mice But Predisposes Neurons to Oxidative Stress. J. Neurosci..

[61] Suraweera A., Lim Y., Woods R., Birrell G.W., Nasim T., Becherel O.J., Lavin M.F. (2009). Functional role for senataxin, defective in ataxia oculomotor apraxia type 2, in transcriptional regulation. Hum. Mol. Genet..

[62] Steinmetz E.J., Warren C.L., Kuehner J.N., Panbehi B., Ansari A.Z., Brow D.A. (2006). Genome-Wide Distribution of Yeast RNA Polymerase II and Its Control by Sen1 Helicase. Mol. Cell.

[63] Chen Y.-Z., Bennett C.L., Huynh H.M., Blair I.P., Puls I., Irobi J., Dierick I., Abel A., Kennerson M.L., Rabin B.A. (2004). DNA/RNA Helicase Gene Mutations in a Form of Juvenile Amyotrophic Lateral Sclerosis (ALS4). Am. J. Hum. Genet..

[64] Avemaria F., Lunetta C., Tarlarini C., Mosca L., Maestri E., Marocchi A., Melazzini M., Penco S., Corbo M. (2011). Mutation in the senataxin gene found in a patient affected by familial ALS with juvenile onset and slow progression. Amyotroph. Lateral Scler..

[65] Rudnik-Schöneborn S., Arning L., Epplen J.T., Zerres K. (2012). SETX gene mutation in a family diagnosed autosomal dominant proximal spinal muscular atrophy. Neuromuscul. Disord..

[66] Wu D., Yu W., Kishikawa H., Folkerth R.D., Iafrate A.J., Shen Y., Xin W., Sims K., Hu G. (2007). Angiogenin loss-of-function mutations in amyotrophic lateral sclerosis. Ann. Neurol..

[67] Subramanian V., Crabtree B., Acharya K.R. (2007). Human angiogenin is a neuroprotective factor and amyotrophic lateral sclerosis associated angiogenin variants affect neurite extension/pathfinding and survival of motor neurons. Hum. Mol. Genet..

[68] Tsuji T., Sun Y., Kishimoto K., Olson K.A., Liu S., Hirukawa S., Hu G. (2005). Angiogenin Is Translocated to the Nucleus of HeLa Cells and Is Involved in Ribosomal RNA Transcription and Cell Proliferation. Cancer Res..

[69] Thiyagarajan N., Ferguson R., Subramanian V., Acharya K.R. (2012). Structural and molecular insights into the mechanism of action of human angiogenin-ALS variants in neurons. Nat. Commun..

[70] Kanekura K., Nishimoto I., Aiso S., Matsuoka M. (2006). Characterization of Amyotrophic Lateral Sclerosis-linked P56S Mutation of Vesicle-associated Membrane Protein-associated Protein B (VAPB/ALS8). J. Biol. Chem..

[71] Peretti D., Dahan N., Shimoni E., Hirschberg K., Lev S. (2008). Coordinated Lipid Transfer between the Endoplasmic Reticulum and the Golgi Complex Requires the VAP Proteins and Is Essential for Golgi-mediated Transport. Mol. Biol. Cell.

[72] Tsuda H., Han S.M., Yang Y., Tong C., Lin Y.Q., Mohan K., Haueter C., Zoghbi A., Harati Y., Kwan J. (2008). The Amyotrophic Lateral Sclerosis 8 Protein VAPB Is Cleaved, Secreted, and Acts as a Ligand for Eph Receptors. Cell.

[73] Morotz G.M., De Vos K.J., Vagnoni A., Ackerley S., Shaw C.E., Miller C.C.J. (2012). Amyotrophic lateral sclerosis-associated mutant VAPBP56S perturbs calcium homeostasis to disrupt axonal transport of mitochondria. Hum. Mol. Genet..

[74] Nishimura A.L., Mitne-Neto M., Silva H.C.A., Richieri-Costa A., Middleton S., Cascio D., Kok F., Oliveira J.R.M., Gillingwater T., Webb J. (2004). A Mutation in the Vesicle-Trafficking Protein VAPB Causes Late-Onset Spinal Muscular Atrophy and Amyotrophic Lateral Sclerosis. Am. J. Hum. Genet..

[75] Tran D., Chalhoub A., Schooley A., Zhang W., Ngsee J.K. (2012). A mutation in VAPB that causes amyotrophic lateral sclerosis also causes a nuclear envelope defect. J. Cell Sci..

[76] Qiu L., Qiao T., Beers M., Tan W., Wang H., Yang B., Xu Z. (2013). Widespread aggregation of mutant VAPB associated with ALS does not cause motor neuron degeneration or modulate mutant SOD1 aggregation and toxicity in mice. Mol. Neurodegener..

[77] Van Deerlin V.M., Leverenz J.B., Bekris L.M., Bird T.D., Yuan W., Elman L.B., Clay D., Wood E.M., Chen-Plotkin A.S., Martinez-Lage M. (2008). TARDBP mutations in amyotrophic lateral sclerosis with TDP-43 neuropathology: A genetic and histopathological analysis. Lancet Neurol..

[78] Prasad A., Bharathi V., Sivalingam V., Girdhar A., Patel B.K. (2019). Molecular Mechanisms of TDP-43 Misfolding and Pathology in Amyotrophic Lateral Sclerosis. Front. Mol. Neurosci..

[79] Neumann M., Sampathu D.M., Kwong L.K., Truax A.C., Micsenyi M.C., Chou T.T., Bruce J., Schuck T., Grossman M., Clark C.M. (2006). Ubiquitinated TDP-43 in Frontotemporal Lobar Degeneration and Amyotrophic Lateral Sclerosis. Science.

[80] Ling S.-C., Polymenidou M., Cleveland D.W. (2013). Converging Mechanisms in ALS and FTD: Disrupted RNA and Protein Homeostasis. Neuron.

[81] Rayaprolu S., Fujioka S., Traynor S., Soto-Ortolaza A.I., Petrucelli L., Dickson D.W., Rademakers R., Boylan K.B., Graff-Radford N.R., Uitti R.J. (2013). TARDBP mutations in Parkinson’s disease. Parkinsonism Relat. Disord..

[82] Schwab C., Arai T., Hasegawa M., Yu S., McGeer P.L. (2008). Colocalization of Transactivation-Responsive DNA-Binding Protein 43 and Huntingtin in Inclusions of Huntington Disease. J. Neuropathol. Exp. Neurol..

[83] King A., Sweeney F., Bodi I., Troakes C., Maekawa S., Al-Sarraj S. (2010). Abnormal TDP-43 expression is identified in the neocortex in cases of dementia pugilistica, but is mainly confined to the limbic system when identified in high and moderate stages of Alzheimer’s disease. Neuropathology.

[84] Nakashima-Yasuda H., Uryu K., Robinson J., Xie S.X., Hurtig H., Duda J.E., Arnold S.E., Siderowf A., Grossman M., Leverenz J.B. (2007). Co-morbidity of TDP-43 proteinopathy in Lewy body related diseases. Acta Neuropathol..

[85] Kwiatkowski T.J., Bosco D.A., Leclerc A.L., Tamrazian E., Vanderburg C.R., Russ C., Davis A., Gilchrist J., Kasarskis E.J., Munsat T. (2009). Mutations in the FUS/TLS gene on chromosome 16 cause familial amyotrophic lateral sclerosis. Science.

[86] Chow C.Y., Landers J.E., Bergren S.K., Sapp P.C., Grant A.E., Jones J.M., Everett L., Lenk G.M., McKenna-Yasek D.M., Weisman L.S. (2009). Deleterious Variants of FIG4, a Phosphoinositide Phosphatase, in Patients with ALS. Am. J. Hum. Genet..

[87] Lagier-Tourenne C., Polymenidou M., Cleveland D.W. (2010). TDP-43 and FUS/TLS: Emerging roles in RNA processing and neurodegeneration. Hum. Mol. Genet..

[88] Vandoorne T., Veys K., Guo W., Sicart A., Vints K., Swijsen A., Moisse M., Eelen G., Gounko N.V., Fumagalli L. (2019). Differentiation but not ALS mutations in FUS rewires motor neuron metabolism. Nat. Commun..

[89] Shelkovnikova T.A., Peters O.M., Deykin A.V., Connor-Robson N., Robinson H., Ustyugov A.A., Bachurin S.O., Ermolkevich T.G., Goldman I.L., Sadchikova E.R. (2013). Fused in Sarcoma (FUS) Protein Lacking Nuclear Localization Signal (NLS) and Major RNA Binding Motifs Triggers Proteinopathy and Severe Motor Phenotype in Transgenic Mice. J. Biol. Chem..

[90] Gentile F., Scarlino S., Falzone Y.M., Lunetta C., Tremolizzo L., Quattrini A., Riva N. (2019). The Peripheral Nervous System in Amyotrophic Lateral Sclerosis: Opportunities for Translational Research. Front. Neurosci..

[91] Chow C.Y., Zhang Y., Dowling J.J., Jin N., Adamska M., Shiga K., Szigeti K., Shy M.E., Li J., Zhang X. (2007). Mutation of FIG4 causes neurodegeneration in the pale tremor mouse and patients with CMT4J. Nature.

[92] Tsai C.-P., Soong B.-W., Lin K.-P., Tu P.-H., Lin J.-L., Lee Y.-C. (2011). FUS, TARDBP, and SOD1 mutations in a Taiwanese cohort with familial ALS. Neurobiol. Aging.

[93] Verdiani S., Origone P., Geroldi A., Bandettini Di Poggio M., Mantero V., Bellone E., Mancardi G., Caponnetto C., Mandich P. (2013). The FIG4 gene does not play a major role in causing ALS in Italian patients. Amyotroph. Lateral Scler. Front. Degener..

[94] Maruyama H., Morino H., Ito H., Izumi Y., Kato H., Watanabe Y., Kinoshita Y., Kamada M., Nodera H., Suzuki H. (2010). Mutations of optineurin in amyotrophic lateral sclerosis. Nature.

[95] Orlacchio A., Babalini C., Borreca A., Patrono C., Massa R., Basaran S., Munhoz R.P., Rogaeva E.A., St George-Hyslop P.H., Bernardi G. (2010). SPATACSIN mutations cause autosomal recessive juvenile amyotrophic lateral sclerosis. Brain.

[96] Liao S., Shen L., Du J., Zhao G., Wang X., Yang Y., Xiao Z., Yuan Y., Jiang H., Li N. (2008). Novel mutations of the SPG11 gene in hereditary spastic paraplegia with thin corpus callosum. J. Neurol. Sci..

[97] Branchu J., Boutry M., Sourd L., Depp M., Leone C., Corriger A., Vallucci M., Esteves T., Matusiak R., Dumont M. (2017). Loss of spatacsin function alters lysosomal lipid clearance leading to upper and lower motor neuron degeneration. Neurobiol. Dis..

[98] Elden A.C., Kim H.-J., Hart M.P., Chen-Plotkin A.S., Johnson B.S., Fang X., Armakola M., Geser F., Greene R., Lu M.M. (2010). Ataxin-2 intermediate-length polyglutamine expansions are associated with increased risk for ALS. Nature.

[99] Fittschen M., Lastres-Becker I., Halbach M.V., Damrath E., Gispert S., Azizov M., Walter M., Müller S., Auburger G. (2015). Genetic ablation of ataxin-2 increases several global translation factors in their transcript abundance but decreases translation rate. Neurogenetics.

[100] Satterfield T.F., Pallanck L.J. (2006). Ataxin-2 and its Drosophila homolog, ATX2, physically assemble with polyribosomes. Hum. Mol. Genet..

[101] Van Damme P., Veldink J.H., van Blitterswijk M., Corveleyn A., van Vught P.W.J., Thijs V., Dubois B., Matthijs G., van den Berg L.H., Robberecht W. (2011). Expanded ATXN2 CAG repeat size in ALS identifies genetic overlap between ALS and SCA2. Neurology.

[102] Liu X., Lu M., Tang L., Zhang N., Chui D., Fan D. (2013). ATXN2 CAG repeat expansions increase the risk for Chinese patients with amyotrophic lateral sclerosis. Neurobiol. Aging.

[103] Meyer H., Bug M., Bremer S. (2012). Emerging functions of the VCP/p97 AAA-ATPase in the ubiquitin system. Nat. Cell Biol..

[104] Ju J.-S., Fuentealba R.A., Miller S.E., Jackson E., Piwnica-Worms D., Baloh R.H., Weihl C.C. (2009). Valosin-containing protein (VCP) is required for autophagy and is disrupted in VCP disease. J. Cell Biol..

[105] Verma R., Oania R.S., Kolawa N.J., Deshaies R.J. (2013). Cdc48/p97 promotes degradation of aberrant nascent polypeptides bound to the ribosome. Elife.

[106] Rumpf S., Bagley J.A., Thompson-Peer K.L., Zhu S., Gorczyca D., Beckstead R.B., Jan L.Y., Jan Y.N. (2014). Drosophila Valosin-Containing Protein is required for dendrite pruning through a regulatory role in mRNA metabolism. Proc. Natl. Acad. Sci. USA.

[107] Johnson J.O., Mandrioli J., Benatar M., Abramzon Y., Van Deerlin V.M., Trojanowski J.Q., Gibbs J.R., Brunetti M., Gronka S., Wuu J. (2010). Exome Sequencing Reveals VCP Mutations as a Cause of Familial ALS. Neuron.

[108] Yin H.Z., Nalbandian A., Hsu C.-I., Li S., Llewellyn K.J., Mozaffar T., Kimonis V.E., Weiss J.H. (2012). Slow development of ALS-like spinal cord pathology in mutant valosin-containing protein gene knock-in mice. Cell Death Dis..

[109] Williams K.L., Warraich S.T., Yang S., Solski J.A., Fernando R., Rouleau G.A., Nicholson G.A., Blair I.P. (2012). UBQLN2/ubiquilin 2 mutation and pathology in familial amyotrophic lateral sclerosis. Neurobiol. Aging.

[110] Goutman S.A., Chen K.S., Paez-Colasante X., Feldman E.L. (2018). Emerging understanding of the genotype–phenotype relationship in amyotrophic lateral sclerosis. Handb. Clin. Neurol..

[111] Mizuno Y., Amari M., Takatama M., Aizawa H., Mihara B., Okamoto K. (2006). Immunoreactivities of p62, an ubiqutin-binding protein, in the spinal anterior horn cells of patients with amyotrophic lateral sclerosis. J. Neurol. Sci..

[112] DeJesus-Hernandez M., Mackenzie I.R.R., Boeve B.F.F., Boxer A.L.L., Baker M., Rutherford N.J.J., Nicholson A.M.M., Finch N.A.A., Flynn H., Adamson J. (2011). Expanded GGGGCC hexanucleotide repeat in noncoding region of C9ORF72 causes chromosome 9p-linked FTD and ALS. Neuron.

[113] Renton A.E., Majounie E., Waite A., Simón-Sánchez J., Rollinson S., Gibbs J.R., Schymick J.C., Laaksovirta H., van Swieten J.C., Myllykangas L. (2011). A Hexanucleotide Repeat Expansion in C9ORF72 Is the Cause of Chromosome 9p21-Linked ALS-FTD. Neuron.

[114] Umoh M.E., Fournier C., Li Y., Polak M., Shaw L., Landers J.E., Hu W., Gearing M., Glass J.D. (2016). Comparative analysis of C9orf72 and sporadic disease in an ALS clinic population. Neurology.

[115] Iacoangeli A., Al Khleifat A., Jones A.R., Sproviero W., Shatunov A., Opie-Martin S., Morrison K.E., Shaw P.J., Shaw C.E., Fogh I. (2019). C9orf72 intermediate expansions of 24–30 repeats are associated with ALS. Acta Neuropathol. Commun..

[116] Balendra R., Isaacs A.M. (2018). C9orf72-mediated ALS and FTD: Multiple pathways to disease. Nat. Rev. Neurol..

[117] Cooper-Knock J., Frolov A., Highley J.R., Charlesworth G., Kirby J., Milano A., Hartley J., Ince P.G., McDermott C.J., Lashley T. (2013). C9ORF72 expansions, parkinsonism, and Parkinson disease: A clinicopathologic study. Neurology.

[118] Devenney E.M., Ahmed R.M., Halliday G., Piguet O., Kiernan M.C., Hodges J.R. (2018). Psychiatric disorders in C9orf72 kindreds. Neurology.

[119] Wu C.-H., Fallini C., Ticozzi N., Keagle P.J., Sapp P.C., Piotrowska K., Lowe P., Koppers M., McKenna-Yasek D., Baron D.M. (2012). Mutations in the profilin 1 gene cause familial amyotrophic lateral sclerosis. Nature.

[120] Yang C., Danielson E.W., Qiao T., Metterville J., Brown R.H., Landers J.E., Xu Z. (2016). Mutant PFN1 causes ALS phenotypes and progressive motor neuron degeneration in mice by a gain of toxicity. Proc. Natl. Acad. Sci. USA.

[121] Kim H.J., Kim N.C., Wang Y.-D., Scarborough E.A., Moore J., Diaz Z., MacLea K.S., Freibaum B., Li S., Molliex A. (2013). Mutations in prion-like domains in hnRNPA2B1 and hnRNPA1 cause multisystem proteinopathy and ALS. Nature.

[122] Honda H., Hamasaki H., Wakamiya T., Koyama S., Suzuki S.O., Fujii N., Iwaki T. (2015). Loss of hnRNPA1 in ALS spinal cord motor neurons with TDP-43-positive inclusions. Neuropathology.

[123] Smith B.N., Ticozzi N., Fallini C., Gkazi A.S., Topp S., Kenna K.P., Scotter E.L., Kost J., Keagle P., Miller J.W. (2014). Exome-wide Rare Variant Analysis Identifies TUBA4A Mutations Associated with Familial ALS. Neuron.

[124] Perrone F., Nguyen H.P., Van Mossevelde S., Moisse M., Sieben A., Santens P., De Bleecker J., Vandenbulcke M., Engelborghs S., Baets J. (2017). Investigating the role of ALS genes CHCHD10 and TUBA4A in Belgian FTD-ALS spectrum patients. Neurobiol. Aging.

[125] Li J., He J., Tang L., Chen L., Ma Y., Fan D. (2018). Screening for TUBA4A mutations in a large Chinese cohort of patients with ALS: Re-evaluating the pathogenesis of TUBA4A in ALS. J. Neurol. Neurosurg. Psychiatry.

[126] Johnson J.O., Pioro E.P., Boehringer A., Chia R., Feit H., Renton A.E., Pliner H.A., Abramzon Y., Marangi G., Winborn B.J. (2014). Mutations in the Matrin 3 gene cause familial amyotrophic lateral sclerosis. Nat. Neurosci..

[127] Salton M., Elkon R., Borodina T., Davydov A., Yaspo M.-L., Halperin E., Shiloh Y. (2011). Matrin 3 Binds and Stabilizes mRNA. PLoS ONE.

[128] Coelho M.B., Attig J., Bellora N., König J., Hallegger M., Kayikci M., Eyras E., Ule J., Smith C.W. (2015). Nuclear matrix protein Matrin3 regulates alternative splicing and forms overlapping regulatory networks with PTB. EMBO J..

[129] Boehringer A., Garcia-Mansfield K., Singh G., Bakkar N., Pirrotte P., Bowser R. (2017). ALS Associated Mutations in Matrin 3 Alter Protein-Protein Interactions and Impede mRNA Nuclear Export. Sci. Rep..

[130] Cirulli E.T., Lasseigne B.N., Petrovski S., Sapp P.C., Dion P.A., Leblond C.S., Couthouis J., Lu Y.-F., Wang Q., Krueger B.J. (2015). Exome sequencing in amyotrophic lateral sclerosis identifies risk genes and pathways. Science.

[131] Brenner D., Müller K., Wieland T., Weydt P., Böhm S., Lulé D., Hübers A., Neuwirth C., Weber M., Borck G. (2016). NEK1 mutations in familial amyotrophic lateral sclerosis. Brain.

[132] Kenna K.P., van Doormaal P.T.C., Dekker A.M., Ticozzi N., Kenna B.J., Diekstra F.P., van Rheenen W., van Eijk K.R., Jones A.R., Keagle P. (2016). NEK1 variants confer susceptibility to amyotrophic lateral sclerosis. Nat. Genet..

[133] van Rheenen W., Shatunov A., Dekker A.M., McLaughlin R.L., Diekstra F.P., Pulit S.L., van der Spek R.A.A., Võsa U., de Jong S., Robinson M.R. (2016). Genome-wide association analyses identify new risk variants and the genetic architecture of amyotrophic lateral sclerosis. Nat. Genet..

[134] Freischmidt A., Wieland T., Richter B., Ruf W., Schaeffer V., Müller K., Marroquin N., Nordin F., Hübers A., Weydt P. (2015). Haploinsufficiency of TBK1 causes familial ALS and fronto-temporal dementia. Nat. Neurosci..

[135] Williams K.L., Topp S., Yang S., Smith B., Fifita J.A., Warraich S.T., Zhang K.Y., Farrawell N., Vance C., Hu X. (2016). CCNF mutations in amyotrophic lateral sclerosis and frontotemporal dementia. Nat. Commun..

[136] Yu Y., Nakagawa T., Morohoshi A., Nakagawa M., Ishida N., Suzuki N., Aoki M., Nakayama K. (2019). Pathogenic mutations in the ALS gene CCNF cause cytoplasmic mislocalization of Cyclin F and elevated VCP ATPase activity. Hum. Mol. Genet..

[137] Nicolas A., Kenna K.P., Renton A.E., Ticozzi N., Faghri F., Chia R., Dominov J.A., Kenna B.J., Nalls M.A., Keagle P. (2018). Genome-wide Analyses Identify KIF5A as a Novel ALS Gene. Neuron.

[138] Campbell P.D., Shen K., Sapio M.R., Glenn T.D., Talbot W.S., Marlow F.L. (2014). Unique Function of Kinesin Kif5A in Localization of Mitochondria in Axons. J. Neurosci..

[139] Hancock W.O., Howard J. (1998). Processivity of the Motor Protein Kinesin Requires Two Heads. J. Cell Biol..

[140] Liu Y.-T., Laura M., Hersheson J., Horga A., Jaunmuktane Z., Brandner S., Pittman A., Hughes D., Polke J.M., Sweeney M.G. (2014). Extended phenotypic spectrum of KIF5A mutations: From spastic paraplegia to axonal neuropathy. Neurology.

[141] Gu X., Li C., Chen Y., Wei Q., Cao B., Ou R., Yuan X., Hou Y., Zhang L., Liu H. (2019). Mutation screening of the KIF5A gene in Chinese patients with amyotrophic lateral sclerosis. J. Neurol. Neurosurg. Psychiatry.

[142] Filosto M., Piccinelli S., Palmieri I., Necchini N., Valente M., Zanella I., Biasiotto G., Lorenzo D., Cereda C., Padovani A. (2018). A Novel Mutation in the Stalk Domain of KIF5A Causes a Slowly Progressive Atypical Motor Syndrome. J. Clin. Med..

[143] Brenner D., Yilmaz R., Müller K., Grehl T., Petri S., Meyer T., Grosskreutz J., Weydt P., Ruf W., Neuwirth C. (2018). Hot-spot KIF5A mutations cause familial ALS. Brain.

[144] Faber I., Martinez A.R.M., de Rezende T.J.R., Martins C.R., Martins M.P., Lourenço C.M., Marques W., Montecchiani C., Orlacchio A., Pedroso J.L. (2018). SPG11 mutations cause widespread white matter and basal ganglia abnormalities, but restricted cortical damage. NeuroImage Clin..

[145] Liu Z.-J., Lin H.-X., Liu G.-L., Tao Q.-Q., Ni W., Xiao B.-G., Wu Z.-Y. (2017). The investigation of genetic and clinical features in Chinese patients with juvenile amyotrophic lateral sclerosis. Clin. Genet..

[146] Zou Z.-Y., Cui L.-Y., Sun Q., Li X.-G., Liu M.-S., Xu Y., Zhou Y., Yang X.-Z. (2013). De novo FUS gene mutations are associated with juvenile-onset sporadic amyotrophic lateral sclerosis in China. Neurobiol. Aging.

[147] Mackenzie I.R.A., Ansorge O., Strong M., Bilbao J., Zinman L., Ang L.-C., Baker M., Stewart H., Eisen A., Rademakers R. (2011). Pathological heterogeneity in amyotrophic lateral sclerosis with FUS mutations: Two distinct patterns correlating with disease severity and mutation. Acta Neuropathol..

[148] Waibel S., Neumann M., Rosenbohm A., Birve A., Volk A.E., Weishaupt J.H., Meyer T., Müller U., Andersen P.M., Ludolph A.C. (2013). Truncating mutations in FUS/TLS give rise to a more aggressive ALS-phenotype than missense mutations: A clinico-genetic study in Germany. Eur. J. Neurol..

[149] Orban P., Devon R.S., Hayden M.R., Leavitt B.R. (2007). Chapter 15 Juvenile amyotrophic lateral sclerosis. Handbook of Clinical Neurology.

[150] Zhao Z., Chen W., Wu Z., Wang N., Zhao G., Chen W., Murong S. (2009). A novel mutation in the senataxin gene identified in a Chinese patient with sporadic amyotrophic lateral sclerosis. Amyotroph. Lateral Scler..

[151] Murphy N.A., Arthur K.C., Tienari P.J., Houlden H., Chiò A., Traynor B.J. (2017). Age-related penetrance of the C9orf72 repeat expansion. Sci. Rep..

[152] Trojsi F., Siciliano M., Femiano C., Santangelo G., Lunetta C., Calvo A., Moglia C., Marinou K., Ticozzi N., Ferro C. (2019). Comparative Analysis of C9orf72 and Sporadic Disease in a Large Multicenter ALS Population: The Effect of Male Sex on Survival of C9orf72 Positive Patients. Front. Neurosci..

[153] Rooney J., Fogh I., Westeneng H.-J., Vajda A., McLaughlin R., Heverin M., Jones A., van Eijk R., Calvo A., Mazzini L. (2017). C9orf72 expansion differentially affects males with spinal onset amyotrophic lateral sclerosis. J. Neurol. Neurosurg. Psychiatry.

[154] Liu K.X., Edwards B., Lee S., Finelli M.J., Davies B., Davies K.E., Oliver P.L. (2015). Neuron-specific antioxidant OXR1 extends survival of a mouse model of amyotrophic lateral sclerosis. Brain.

[155] Fischer L.R., Culver D.G., Tennant P., Davis A.A., Wang M., Castellano-Sanchez A., Khan J., Polak M.A., Glass J.D. (2004). Amyotrophic lateral sclerosis is a distal axonopathy: Evidence in mice and man. Exp. Neurol..

[156] Park J.H., Elpers C., Reunert J., McCormick M.L., Mohr J., Biskup S., Schwartz O., Rust S., Grüneberg M., Seelhöfer A. (2019). SOD1 deficiency: A novel syndrome distinct from amyotrophic lateral sclerosis. Brain.

[157] Tasca G., Lattante S., Marangi G., Conte A., Bernardo D., Bisogni G., Mandich P., Zollino M., Ragozzino E., Udd B. (2020). SOD1 p.D12Y variant is associated with ALS/distal myopathy spectrum. Eur. J. Neurol..

[158] Stewart H., Rutherford N.J., Briemberg H., Krieger C., Cashman N., Fabros M., Baker M., Fok A., DeJesus-Hernandez M., Eisen A. (2012). Clinical and pathological features of amyotrophic lateral sclerosis caused by mutation in the C9ORF72 gene on chromosome 9p. Acta Neuropathol..

[159] Canosa A., Calvo A., Moglia C., Barberis M., Brunetti M., Cammarosano S., Manera U., Ilardi A., Restagno G., Chiò A. (2015). A novel p.E121G heterozygous missense mutation of SOD1 in an apparently sporadic ALS case with a 14-year course. Amyotroph. Lateral Scler. Front. Degener..

[160] Corcia P., Vourc’h P., Blasco H., Couratier P., Dangoumau A., Bellance R., Desnuelle C., Viader F., Pautot V., Millecamps S. (2018). Phenotypic and genotypic studies of ALS cases in ALS-SMA families. Amyotroph. Lateral Scler. Front. Degener..

[161] Battistini S., Ricci C., Giannini F., Calzavara S., Greco G., Del Corona A., Mancuso M., Battistini N., Siciliano G., Carrera P. (2010). G41S SOD1 mutation: A common ancestor for six ALS Italian families with an aggressive phenotype. Amyotroph. Lateral Scler..

[162] Nizzardo M., Simone C., Rizzo F., Ulzi G., Ramirez A., Rizzuti M., Bordoni A., Bucchia M., Gatti S., Bresolin N. (2016). Morpholino-mediated SOD1 reduction ameliorates an amyotrophic lateral sclerosis disease phenotype. Sci. Rep..

[163] Rohrer J.D., Isaacs A.M., Mizielinska S., Mead S., Lashley T., Wray S., Sidle K., Fratta P., Orrell R.W., Hardy J. (2015). C9orf72 expansions in frontotemporal dementia and amyotrophic lateral sclerosis. Lancet Neurol..

[164] Le Ber I. (2013). SQSTM1 Mutations in French Patients With Frontotemporal Dementia or Frontotemporal Dementia With Amyotrophic Lateral Sclerosis. JAMA Neurol..

[165] Lamb R., Rohrer J.D., Real R., Lubbe S.J., Waite A.J., Blake D.J., Walters R.J., Lashley T., Revesz T., Holton J.L. (2019). A novel TBK1 mutation in a family with diverse frontotemporal dementia spectrum disorders. Mol. Case Stud..

[166] Blauwendraat C., Wilke C., Simón-Sánchez J., Jansen I.E., Reifschneider A., Capell A., Haass C., Castillo-Lizardo M., Biskup S., Maetzler W. (2018). The wide genetic landscape of clinical frontotemporal dementia: Systematic combined sequencing of 121 consecutive subjects. Genet. Med..

[167] Chia R., Chiò A., Traynor B.J. (2018). Novel genes associated with amyotrophic lateral sclerosis: Diagnostic and clinical implications. Lancet Neurol..

[168] Liu Q., Shu S., Wang R.R., Liu F., Cui B., Guo X.N., Lu C.X., Li X.G., Liu M.S., Peng B. (2016). Whole-exome sequencing identifies a missense mutation in hnRNPA1 in a family with flail arm ALS. Neurology.

[169] Tripolszki K., Török D., Goudenège D., Farkas K., Sulák A., Török N., Engelhardt J.I., Klivényi P., Procaccio V., Nagy N. (2017). High-throughput sequencing revealed a novel SETX mutation in a Hungarian patient with amyotrophic lateral sclerosis. Brain Behav..

[170] Kenna K.P., McLaughlin R.L., Byrne S., Elamin M., Heverin M., Kenny E.M., Cormican P., Morris D.W., Donaghy C.G., Bradley D.G. (2013). Delineating the genetic heterogeneity of ALS using targeted high-throughput sequencing. J. Med. Genet..

[171] Cady J., Allred P., Bali T., Pestronk A., Goate A., Miller T.M., Mitra R.D., Ravits J., Harms M.B., Baloh R.H. (2015). Amyotrophic lateral sclerosis onset is influenced by the burden of rare variants in known amyotrophic lateral sclerosis genes. Ann. Neurol..

[172] Liu Z.-J., Lin H.-X., Wei Q., Zhang Q.-J., Chen C.-X., Tao Q.-Q., Liu G.-L., Ni W., Gitler A.D., Li H.-F. (2019). Genetic Spectrum and Variability in Chinese Patients with Amyotrophic Lateral Sclerosis. Aging Dis..

[173] Tsai Y., Lin K., Jih K., Tsai P., Liao Y., Lee Y. (2020). Hand-onset weakness is a common feature of ALS patients with a NEK1 loss-of-function variant. Ann. Clin. Transl. Neurol..

[174] Ricci C., Giannini F., Intini E., Battistini S. (2019). Genotype–phenotype correlation and evidence for a common ancestor in two Italian ALS patients with the D124G SOD1 mutation. Amyotroph. Lateral Scler. Front. Degener..

[175] Dalla Bella E., Lombardi R., Porretta-Serapiglia C., Ciano C., Gellera C., Pensato V., Cazzato D., Lauria G. (2016). Amyotrophic lateral sclerosis causes small fiber pathology. Eur. J. Neurol..

[176] Khani M., Alavi A., Nafissi S., Elahi E. (2015). Observation of c.260A > G mutation in superoxide dismutase 1 that causes p.Asn86Ser in Iranian amyotrophic lateral sclerosis patient and absence of genotype/phenotype correlation. Iran. J. Neurol..

[177] Chiò A., Mora G., Sabatelli M., Caponnetto C., Lunetta C., Traynor B.J., Johnson J.O., Nalls M.A., Calvo A., Moglia C. (2015). HFE p.H63D polymorphism does not influence ALS phenotype and survival. Neurobiol. Aging.

[178] Kim M.-J., Bae J.-H., Kim J.-M., Kim H.R., Yoon B.-N., Sung J.-J., Ahn S.-W. (2016). Rapid Progression of Sporadic ALS in a Patient Carrying SOD1 p.Gly13Arg Mutation. Exp. Neurobiol..

[179] Chen W., Xie Y., Zheng M., Lin J., Huang P., Pei Z., Yao X. (2020). Clinical and genetic features of patients with amyotrophic lateral sclerosis in southern China. Eur. J. Neurol..

[180] Lattante S., Conte A., Zollino M., Luigetti M., Del Grande A., Marangi G., Romano A., Marcaccio A., Meleo E., Bisogni G. (2012). Contribution of major amyotrophic lateral sclerosis genes to the etiology of sporadic disease. Neurology.

[181] Felbecker A., Camu W., Valdmanis P.N., Sperfeld A.D., Waibel S., Steinbach P., Rouleau G.A., Ludolph A.C., Andersen P.M. (2010). Four familial ALS pedigrees discordant for two SOD1 mutations: Are all SOD1 mutations pathogenic?. J. Neurol. Neurosurg. Psychiatry.

[182] Nogales-Gadea G., Garcia-Arumi E., Andreu A.L., Cervera C., Gamez J. (2004). A novel exon 5 mutation (N139H) in the SOD1 gene in a Spanish family associated with incomplete penetrance. J. Neurol. Sci..

[183] Ferrera L., Caponnetto C., Marini V., Rizzi D., Bordo D., Penco S., Amoroso A., Origone P., Garrè C. (2003). An Italian dominant FALS Leu144Phe SOD1 mutation: Genotype-phenotype correlation. Amyotroph. Lateral Scler. Other Mot. Neuron Disord..

[184] Luisa Conforti F., Sprovieri T., Mazzei R., Patitucci A., Ungaro C., Zoccolella S., Magariello A., Bella V.L., Tessitore A., Tedeschi G. (2009). Further evidence that D90A-SOD1 mutation is recessively inherited in ALS patients in Italy. Amyotroph. Lateral Scler..

[185] Murakami T., Warita H., Hayashi T., Sato K., Manabe Y., Mizuno S., Yamane K., Abe K. (2001). A novel SOD1 gene mutation in familial ALS with low penetrance in females. J. Neurol. Sci..

[186] Segovia-Silvestre T., Andreu A.L., Vives-Bauza C., Garcia-Arumi E., Cervera C., Gamez J. (2002). A novel exon 3 mutation (D76V) in the SOD1 gene associated with slowly progressive ALS. Amyotroph. Lateral Scler. Other Mot. Neuron Disord..

[187] Ticozzi N., Tiloca C., Mencacci N.E., Morelli C., Doretti A., Rusconi D., Colombrita C., Sangalli D., Verde F., Finelli P. (2013). Oligoclonal bands in the cerebrospinal fluid of amyotrophic lateral sclerosis patients with disease-associated mutations. J. Neurol..

[188] Mandrioli J., Michalke B., Solovyev N., Grill P., Violi F., Lunetta C., Conte A., Sansone V.A., Sabatelli M., Vinceti M. (2017). Elevated Levels of Selenium Species in Cerebrospinal Fluid of Amyotrophic Lateral Sclerosis Patients with Disease-Associated Gene Mutations. Neurodegener. Dis..

[189] van der Zee J., Gijselinck I., Van Mossevelde S., Perrone F., Dillen L., Heeman B., Bäumer V., Engelborghs S., De Bleecker J., Baets J. (2017). TBK1 Mutation Spectrum in an Extended European Patient Cohort with Frontotemporal Dementia and Amyotrophic Lateral Sclerosis. Hum. Mutat..

[190] Weinreich M., Shepheard S.R., Verber N., Wyles M., Heath P.R., Highley J.R., Kirby J., Shaw P.J. (2019). Neuropathological characterization of a novel TANK binding kinase (TBK1) gene loss of function mutation associated with amyotrophic lateral sclerosis. Neuropathol. Appl. Neurobiol..

[191] Caroppo P., Camuzat A., De Septenville A., Couratier P., Lacomblez L., Auriacombe S., Flabeau O., Jornéa L., Blanc F., Sellal F. (2015). Semantic and nonfluent aphasic variants, secondarily associated with amyotrophic lateral sclerosis, are predominant frontotemporal lobar degeneration phenotypes in TBK1 carriers. Alzheimer’s Dement. Diagnosis, Assess. Dis. Monit..

[192] Dols-Icardo O., García-Redondo A., Rojas-García R., Borrego-Hernández D., Illán-Gala I., Muñoz-Blanco J.L., Rábano A., Cervera-Carles L., Juárez-Rufián A., Spataro N. (2018). Analysis of known amyotrophic lateral sclerosis and frontotemporal dementia genes reveals a substantial genetic burden in patients manifesting both diseases not carrying the C9orf72 expansion mutation. J. Neurol. Neurosurg. Psychiatry.

[193] Calvo A., Moglia C., Canosa A., Brunetti M., Barberis M., Traynor B.J., Carrara G., Valentini C., Restagno G., Chiò A. (2014). A de novo nonsense mutation of the FUS gene in an apparently familial amyotrophic lateral sclerosis case. Neurobiol. Aging.

[194] Damme P.V., Goris A., Race V., Hersmus N., Dubois B., Bosch L.V.D., Matthijs G., Robberecht W. (2010). The occurrence of mutations in FUS in a Belgian cohort of patients with familial ALS. Eur. J. Neurol..

[195] Zou Z.-Y., Liu M.-S., Li X.-G., Cui L.-Y. (2016). Mutations in FUS are the most frequent genetic cause in juvenile sporadic ALS patients of Chinese origin. Amyotroph. Lateral Scler. Front. Degener..

[196] Naumann M., Peikert K., Günther R., Kooi A.J., Aronica E., Hübers A., Danel V., Corcia P., Pan-Montojo F., Cirak S. (2019). Phenotypes and malignancy risk of different FUS mutations in genetic amyotrophic lateral sclerosis. Ann. Clin. Transl. Neurol..

[197] Tümer Z., Bertelsen B., Gredal O., Magyari M., Nielsen K.C., Grønskov K., Brøndum-Nielsen K., LuCamp (2012). A novel heterozygous nonsense mutation of the OPTN gene segregating in a Danish family with ALS. Neurobiol. Aging.

[198] Iida A., Hosono N., Sano M., Kamei T., Oshima S., Tokuda T., Nakajima M., Kubo M., Nakamura Y., Ikegawa S. (2012). Novel deletion mutations of OPTN in amyotrophic lateral sclerosis in Japanese. Neurobiol. Aging.

[199] Feng S., Che C., Feng S., Liu C., Li L., Li Y., Huang H., Zou Z. (2019). Novel mutation in optineurin causing aggressive ALS+/−frontotemporal dementia. Ann. Clin. Transl. Neurol..

[200] Li C., Ji Y., Tang L., Zhang N., He J., Ye S., Liu X., Fan D. (2015). Optineurin mutations in patients with sporadic amyotrophic lateral sclerosis in China. Amyotroph. Lateral Scler. Front. Degener..

[201] Goldstein O., Nayshool O., Nefussy B., Traynor B.J., Renton A.E., Gana-Weisz M., Drory V.E., Orr-Urtreger A. (2016). OPTN 691_692insAG is a founder mutation causing recessive ALS and increased risk in heterozygotes. Neurology.

[202] Weishaupt J.H., Waibel S., Birve A., Volk A.E., Mayer B., Meyer T., Ludolph A.C., Andersen P.M. (2013). A novel optineurin truncating mutation and three glaucoma-associated missense variants in patients with familial amyotrophic lateral sclerosis in Germany. Neurobiol. Aging.

[203] Del Bo R., Tiloca C., Pensato V., Corrado L., Ratti A., Ticozzi N., Corti S., Castellotti B., Mazzini L., Soraru G. (2011). Novel optineurin mutations in patients with familial and sporadic amyotrophic lateral sclerosis. J. Neurol. Neurosurg. Psychiatry.

[204] Li J., He J., Tang L., Chen L., Xu L., Ma Y., Zhang N., Fan D. (2016). TUBA4A may not be a significant genetic factor in Chinese ALS patients. Amyotroph. Lateral Scler. Front. Degener..

[205] Pensato V., Tiloca C., Corrado L., Bertolin C., Sardone V., Del Bo R., Calini D., Mandrioli J., Lauria G., Mazzini L. (2015). TUBA4A gene analysis in sporadic amyotrophic lateral sclerosis: Identification of novel mutations. J. Neurol..

[206] Borghero G., Pugliatti M., Marrosu F., Marrosu M.G., Murru M.R., Floris G., Cannas A., Parish L.D., Occhineri P., Cau T.B. (2014). Genetic architecture of ALS in Sardinia. Neurobiol. Aging.

[207] Corcia P., Valdmanis P., Millecamps S., Lionnet C., Blasco H., Mouzat K., Daoud H., Belzil V., Morales R., Pageot N. (2012). Phenotype and genotype analysis in amyotrophic lateral sclerosis with TARDBP gene mutations. Neurology.

[208] Orrù S., Manolakos E., Orrù N., Kokotas H., Mascia V., Carcassi C., Petersen M.B. (2012). High frequency of the TARDBP p.Ala382Thr mutation in Sardinian patients with amyotrophic lateral sclerosis. Clin. Genet..

[209] Ticozzi N., LeClerc A.L., van Blitterswijk M., Keagle P., McKenna-Yasek D.M., Sapp P.C., Silani V., Wills A.-M., Brown R.H., Landers J.E. (2011). Mutational analysis of TARDBP in neurodegenerative diseases. Neurobiol. Aging.

[210] Xu G., Hu W., Zhan L.-L., Wang C., Xu L.-Q., Lin M.-T., Chen W.-J., Wang N., Zhang Q.-J. (2018). High frequency of the TARDBP p.M337 V mutation among south-eastern Chinese patients with familial amyotrophic lateral sclerosis. BMC Neurol..

[211] Caroppo P., Camuzat A., Guillot-Noel L., Thomas-Antérion C., Couratier P., Wong T.H., Teichmann M., Golfier V., Auriacombe S., Belliard S. (2016). Defining the spectrum of frontotemporal dementias associated with TARDBP mutations. Neurol. Genet..

[212] Corrado L., Mazzini L., Oggioni G.D., Luciano B., Godi M., Brusco A., D’Alfonso S. (2011). ATXN-2 CAG repeat expansions are interrupted in ALS patients. Hum. Genet..

[213] Tavares de Andrade H.M., Cintra V.P., de Albuquerque M., Piccinin C.C., Bonadia L.C., Duarte Couteiro R.E., Sabino de Oliveira D., Claudino R., Magno Gonçalves M.V., Dourado M.E.T. (2018). Intermediate-length CAG repeat in ATXN2 is associated with increased risk for amyotrophic lateral sclerosis in Brazilian patients. Neurobiol. Aging.

[214] Ross O.A., Rutherford N.J., Baker M., Soto-Ortolaza A.I., Carrasquillo M.M., DeJesus-Hernandez M., Adamson J., Li M., Volkening K., Finger E. (2011). Ataxin-2 repeat-length variation and neurodegeneration. Hum. Mol. Genet..

[215] Millecamps S., Boillée S., Le Ber I., Seilhean D., Teyssou E., Giraudeau M., Moigneu C., Vandenberghe N., Danel-Brunaud V., Corcia P. (2012). Phenotype difference between ALS patients with expanded repeats in C9ORF72 and patients with mutations in other ALS-related genes. J. Med. Genet..

[216] Xi Z., Zinman L., Grinberg Y., Moreno D., Sato C., Bilbao J.M., Ghani M., Hernández I., Ruiz A., Boada M. (2012). Investigation of C9orf72 in 4 Neurodegenerative Disorders. Arch. Neurol..

[217] Dols-Icardo O., Garcia-Redondo A., Rojas-Garcia R., Sanchez-Valle R., Noguera A., Gomez-Tortosa E., Pastor P., Hernandez I., Esteban-Perez J., Suarez-Calvet M. (2014). Characterization of the repeat expansion size in C9orf72 in amyotrophic lateral sclerosis and frontotemporal dementia. Hum. Mol. Genet..

[218] van Blitterswijk M., DeJesus-Hernandez M., Niemantsverdriet E., Murray M.E., Heckman M.G., Diehl N.N., Brown P.H., Baker M.C., Finch N.A., Bauer P.O. (2013). Association between repeat sizes and clinical and pathological characteristics in carriers of C9ORF72 repeat expansions (Xpansize-72): A cross-sectional cohort study. Lancet Neurol..

[219] Gijselinck I., Van Langenhove T., van der Zee J., Sleegers K., Philtjens S., Kleinberger G., Janssens J., Bettens K., Van Cauwenberghe C., Pereson S. (2012). A C9orf72 promoter repeat expansion in a Flanders-Belgian cohort with disorders of the frontotemporal lobar degeneration-amyotrophic lateral sclerosis spectrum: A gene identification study. Lancet Neurol..

[220] Goldstein O., Gana-Weisz M., Nefussy B., Vainer B., Nayshool O., Bar-Shira A., Traynor B.J., Drory V.E., Orr-Urtreger A. (2018). High frequency of C9orf72 hexanucleotide repeat expansion in amyotrophic lateral sclerosis patients from two founder populations sharing the same risk haplotype. Neurobiol. Aging.

[221] Fournier C., Barbier M., Camuzat A., Anquetil V., Lattante S., Clot F., Cazeneuve C., Rinaldi D., Couratier P., Deramecourt V. (2019). Relations between C9orf72 expansion size in blood, age at onset, age at collection and transmission across generations in patients and presymptomatic carriers. Neurobiol. Aging.

[222] Zhang M., Tartaglia M.C., Moreno D., Sato C., McKeever P., Weichert A., Keith J., Robertson J., Zinman L., Rogaeva E. (2017). DNA methylation age-acceleration is associated with disease duration and age at onset in C9orf72 patients. Acta Neuropathol..

[223] Gendron T.F., van Blitterswijk M., Bieniek K.F., Daughrity L.M., Jiang J., Rush B.K., Pedraza O., Lucas J.A., Murray M.E., Desaro P. (2015). Cerebellar c9RAN proteins associate with clinical and neuropathological characteristics of C9ORF72 repeat expansion carriers. Acta Neuropathol..

[224] Kaivorinne A.-L., Bode M.K., Paavola L., Tuominen H., Kallio M., Renton A.E., Traynor B.J., Moilanen V., Remes A.M. (2013). Clinical Characteristics of C9ORF72-Linked Frontotemporal Lobar Degeneration. Dement. Geriatr. Cogn. Dis. Extra.

[225] van der Burgh H.K., Westeneng H.-J., Walhout R., van Veenhuijzen K., Tan H.H.G., Meier J.M., Bakker L.A., Hendrikse J., van Es M.A., Veldink J.H. (2020). Multimodal longitudinal study of structural brain involvement in amyotrophic lateral sclerosis. Neurology.

[226] Floris G., Borghero G., Di Stefano F., Melis R., Puddu R., Fadda L., Murru M.R., Corongiu D., Cuccu S., Tranquilli S. (2016). Phenotypic variability related to C9orf72 mutation in a large Sardinian kindred. Amyotroph. Lateral Scler. Front. Degener..

[227] Narain P., Padhi A.K., Dave U., Mishra D., Bhatia R., Vivekanandan P., Gomes J. (2019). Identification and characterization of novel and rare susceptible variants in Indian amyotrophic lateral sclerosis patients. Neurogenetics.

[228] Origone P., Verdiani S., Bandettini Di Poggio M., Zuccarino R., Vignolo M., Caponnetto C., Mandich P. (2015). A novel Arg147Trp MATR3 missense mutation in a slowly progressive ALS Italian patient. Amyotroph. Lateral Scler. Front. Degener..

[229] Leblond C.S., Gan-Or Z., Spiegelman D., Laurent S.B., Szuto A., Hodgkinson A., Dionne-Laporte A., Provencher P., de Carvalho M., Orrù S. (2016). Replication study of MATR3 in familial and sporadic amyotrophic lateral sclerosis. Neurobiol. Aging.

[230] Lin K.P., Tsai P.C., Liao Y.C., Chen W.T., Tsai C.P., Soong B.W., Lee Y.C. (2015). Mutational analysis of MATR3 in Taiwanese patients with amyotrophic lateral sclerosis. Neurobiol. Aging.

[231] Marangi G., Lattante S., Doronzio P.N., Conte A., Tasca G., Monforte M., Patanella A.K., Bisogni G., Meleo E., La Spada S. (2017). Matrin 3 variants are frequent in Italian ALS patients. Neurobiol. Aging.

[232] Osmanovic A., Rangnau I., Kosfeld A., Abdulla S., Janssen C., Auber B., Raab P., Preller M., Petri S., Weber R.G. (2017). FIG4 variants in central European patients with amyotrophic lateral sclerosis: A whole-exome and targeted sequencing study. Eur. J. Hum. Genet..

[233] Conforti F.L., Sprovieri T., Mazzei R., Ungaro C., La Bella V., Tessitore A., Patitucci A., Magariello A., Gabriele A.L., Tedeschi G. (2008). A novel Angiogenin gene mutation in a sporadic patient with amyotrophic lateral sclerosis from southern Italy. Neuromuscul. Disord..

[234] Paubel A. (2008). Mutations of the ANG Gene in French Patients With Sporadic Amyotrophic Lateral Sclerosis. Arch. Neurol..

[235] Fernández-Santiago R., Hoenig S., Lichtner P., Sperfeld A.-D., Sharma M., Berg D., Weichenrieder O., Illig T., Eger K., Meyer T. (2009). Identification of novel Angiogenin (ANG) gene missense variants in German patients with amyotrophic lateral sclerosis. J. Neurol..

[236] Nguyen H.P., Van Mossevelde S., Dillen L., De Bleecker J.L., Moisse M., Van Damme P., Van Broeckhoven C., van der Zee J., Engelborghs S., Crols R. (2018). NEK1 genetic variability in a Belgian cohort of ALS and ALS-FTD patients. Neurobiol. Aging.

[237] He J., Liu X., Tang L., Zhao C., He J., Fan D. (2020). Whole-exome sequencing identified novel KIF5A mutations in Chinese patients with amyotrophic lateral sclerosis and Charcot-Marie-Tooth type 2. J. Neurol. Neurosurg. Psychiatry.

[238] Tiloca C., Ticozzi N., Pensato V., Corrado L., Del Bo R., Bertolin C., Fenoglio C., Gagliardi S., Calini D., Lauria G. (2013). Screening of the PFN1 gene in sporadic amyotrophic lateral sclerosis and in frontotemporal dementia. Neurobiol. Aging.

[239] Ingre C., Landers J.E., Rizik N., Volk A.E., Akimoto C., Birve A., Hübers A., Keagle P.J., Piotrowska K., Press R. (2013). A novel phosphorylation site mutation in profilin 1 revealed in a large screen of US, Nordic, and German amyotrophic lateral sclerosis/frontotemporal dementia cohorts. Neurobiol. Aging.

[240] Smith B.N., Vance C., Scotter E.L., Troakes C., Wong C.H., Topp S., Maekawa S., King A., Mitchell J.C., Lund K. (2015). Novel mutations support a role for Profilin 1 in the pathogenesis of ALS. Neurobiol. Aging.

[241] Chen Y., Zheng Z.-Z., Huang R., Chen K., Song W., Zhao B., Chen X., Yang Y., Yuan L., Shang H.-F. (2013). PFN1 mutations are rare in Han Chinese populations with amyotrophic lateral sclerosis. Neurobiol. Aging.

[242] van Blitterswijk M., van Es M.A., Koppers M., van Rheenen W., Medic J., Schelhaas H.J., van der Kooi A.J., de Visser M., Veldink J.H., van den Berg L.H. (2012). VAPB and C9orf72 mutations in 1 familial amyotrophic lateral sclerosis patient. Neurobiol. Aging.

[243] Di L., Chen H., Da Y., Wang S., Shen X.-M. (2016). Atypical familial amyotrophic lateral sclerosis with initial symptoms of pain or tremor in a Chinese family harboring VAPB-P56S mutation. J. Neurol..

[244] Gellera C., Tiloca C., Del Bo R., Corrado L., Pensato V., Agostini J., Cereda C., Ratti A., Castellotti B., Corti S. (2013). Ubiquilin 2 mutations in Italian patients with amyotrophic lateral sclerosis and frontotemporal dementia. J. Neurol. Neurosurg. Psychiatry.

[245] Dillen L., Van Langenhove T., Engelborghs S., Vandenbulcke M., Sarafov S., Tournev I., Merlin C., Cras P., Vandenberghe R., De Deyn P.P. (2013). Explorative genetic study of UBQLN2 and PFN1 in an extended Flanders-Belgian cohort of frontotemporal lobar degeneration patients. Neurobiol. Aging.

[246] Yang Y., Tang L., Zhang N., Pan L., Hadano S., Fan D. (2015). Six SQSTM1 mutations in a Chinese amyotrophic lateral sclerosis cohort. Amyotroph. Lateral Scler. Front. Degener..

[247] Al-Obeidi E., Al-Tahan S., Surampalli A., Goyal N., Wang A.K., Hermann A., Omizo M., Smith C., Mozaffar T., Kimonis V. (2018). Genotype-phenotype study in patients with valosin-containing protein mutations associated with multisystem proteinopathy. Clin. Genet..

[248] Daoud H., Zhou S., Noreau A., Sabbagh M., Belzil V., Dionne-Laporte A., Tranchant C., Dion P., Rouleau G.A. (2012). Exome sequencing reveals SPG11 mutations causing juvenile ALS. Neurobiol. Aging.

[249] Cooper-Knock J., Moll T., Ramesh T., Castelli L., Beer A., Robins H., Fox I., Niedermoser I., Van Damme P., Moisse M. (2019). Mutations in the Glycosyltransferase Domain of GLT8D1 Are Associated with Familial Amyotrophic Lateral Sclerosis. Cell Rep..

[250] Dobson-Stone C., Hallupp M., Shahheydari H., Ragagnin A.M.G., Chatterton Z., Carew-Jones F., Shepherd C.E., Stefen H., Paric E., Fath T. (2020). CYLD is a causative gene for frontotemporal dementia—Amyotrophic lateral sclerosis. Brain.

[251] Farhan S.M.K., Howrigan D.P., Abbott L.E., Klim J.R., Topp S.D., Byrnes A.E., Churchhouse C., Phatnani H., Smith B.N., Rampersaud E. (2019). Exome sequencing in amyotrophic lateral sclerosis implicates a novel gene, DNAJC7, encoding a heat-shock protein. Nat. Neurosci..

[252] Cudkowicz M.E., McKenna-Yasek D., Sapp P.E., Chin W., Geller B., Hayden D.L., Schoenfeld D.A., Hosler B.A., Horvitz H.R., Brown R.H. (1997). Epidemiology of mutations in superoxide dismutase in amyotrophic lateal sclerosis. Ann. Neurol..

[253] Ogasawara M., Matsubara Y., Narisawa K., Aoki M., Nakamura S., Itoyama Y., Abe K. (1993). Mild ALS in Japan associated with novel SOD mutation. Nat. Genet..

