# Supplementary material for: A Systematic Review of Genotype–Phenotype Correlation across Cohorts Having Causal Mutations of Different Genes in ALS
Source: J Pers Med. 2020 Jun 29;10(3):58. doi: 10.3390/jpm10030058 (PMC7564886; doi:10.3390/jpm10030058)
Supplement: Supplementary file 1 [file jpm-10-00058-s001.zip › supplementary/Supplemental Material-R.docx]

**Table S1:** Key words used in PubMed literature search with total hits returned and number of studies used to produce Table S2.

| **Key words used** | **Results** | **Included** |
| --- | --- | --- |
| "ALS" AND "Genotype phenotype" AND "Patient" AND "Onset" | 128 | 51 |
| "ALS" AND "Phenotype" AND "Patient" and "TBK1" | 15 | 2 |
| "ALS" AND "Genotype phenotype" AND "Patient" AND "VCP" | 6 | 2 |
| "ALS" AND "Genotype Phenotype" AND "Patient" AND "SQSTM1" | 5 | 1 |
| "ALS" and "Patient" and "CCNF" | 11 | 1 |
| "ALS" AND "NEK1" AND "Cohort" | 9 | 4 |
| "ALS" AND "Phenotype" AND "Patient" AND "OPTN" | 21 | 6 |
| "ALS" AND "Phenotype" AND "Patient" AND "FIG4" | 2 | 2 |
| "ALS" AND "Patient" AND "PFN1" | 26 | 5 |
| "ALS" AND "Phenotype" AND "Patient" AND "ATXN2" | 26 | 5 |
| "ALS" AND "VAPB" AND "Cohort" | 11 | 4 |
| "ALS" AND "Phenotype" AND "Patient" AND "ANG" | 19 | 5 |
| "ALS" AND "Genotype phenotype" AND "Patient" AND "ALS2" | 3 | 1 |
| "ALS" OR "amyotrophic lateral sclerosis[MeSH Terms]" AND "Genotype phenotype" AND "Patient" AND "SPG11" | 3 | 2 |
| "ALS" OR "amyotrophic lateral sclerosis[MeSH Terms]" AND "TUBA4A" | 18 | 4 |
| "ALS" OR "amyotrophic lateral sclerosis[MeSH Terms]" AND "UBQLN2" AND"Patient" AND "Cohort" | 14 | 4 |
| "ALS" OR "amyotrophic lateral sclerosis[MeSH Terms]" AND "KIF5A" | 20 | 4 |
| "ALS OR "amyotrophic lateral sclerosis[MeSH Terms]" AND "Patient" AND "MATR3" | 18 | 4 |

**Table S3:** PRISMA checklist for systematic review.

| **Section/topic** | **#** | **Checklist item** | **Reported on page #** |
| --- | --- | --- | --- |
| **TITLE** | | |  |
| Title | 1 | Identify the report as a systematic review, meta-analysis, or both. | 1 |
| **ABSTRACT** | | |  |
| Structured summary | 2 | Provide a structured summary including, as applicable: background; objectives; data sources; study eligibility criteria, participants, and interventions; study appraisal and synthesis methods; results; limitations; conclusions and implications of key findings; systematic review registration number. | 1 |
| **INTRODUCTION** | | |  |
| Rationale | 3 | Describe the rationale for the review in the context of what is already known. | 1-8 |
| Objectives | 4 | Provide an explicit statement of questions being addressed with reference to participants, interventions, comparisons, outcomes, and study design (PICOS). | 8 |
| **METHODS** | | |  |
| Protocol and registration | 5 | Indicate if a review protocol exists, if and where it can be accessed (e.g., Web address), and, if available, provide registration information including registration number. | Not yet |
| Eligibility criteria | 6 | Specify study characteristics (e.g., PICOS, length of follow-up) and report characteristics (e.g., years considered, language, publication status) used as criteria for eligibility, giving rationale. | 9 |
| Information sources | 7 | Describe all information sources (e.g., databases with dates of coverage, contact with study authors to identify additional studies) in the search and date last searched. | 9 and TableS1 |
| Search | 8 | Present full electronic search strategy for at least one database, including any limits used, such that it could be repeated. | TableS1 |
| Study selection | 9 | State the process for selecting studies (i.e., screening, eligibility, included in systematic review, and, if applicable, included in the meta-analysis). | 9 |
| Data collection process | 10 | Describe method of data extraction from reports (e.g., piloted forms, independently, in duplicate) and any processes for obtaining and confirming data from investigators. | 9-10 |
| Data items | 11 | List and define all variables for which data were sought (e.g., PICOS, funding sources) and any assumptions and simplifications made. | 9 |
| Risk of bias in individual studies | 12 | Describe methods used for assessing risk of bias of individual studies (including specification of whether this was done at the study or outcome level), and how this information is to be used in any data synthesis. | 9 |
| Summary measures | 13 | State the principal summary measures (e.g., risk ratio, difference in means). | 10-13 |
| Synthesis of results | 14 | Describe the methods of handling data and combining results of studies, if done, including measures of consistency (e.g., I^2^) for each meta-analysis. | 10 |
